# An Essential Role for TAGLN2 in Phagocytosis of Lipopolysaccharide-activated Macrophages

**DOI:** 10.1038/s41598-017-09144-x

**Published:** 2017-08-18

**Authors:** Hye-Ran Kim, Hyun-Su Lee, Kyung-Sik Lee, In Duk Jung, Min-Sung Kwon, Chang-Hyun Kim, Seong-Min Kim, Myung-Han Yoon, Yeong-Min Park, Sang-Myeong Lee, Chang-Duk Jun

**Affiliations:** 1 School of Life Sciences, Gwangju, 500-712 Korea; 2 Immune Synapse and Cell Therapy Research Center, GIST, Gwangju, 500-712 Korea; 3 School of Materials Science and Engineering, GIST, Gwangju, 500-712 Korea; 4 Department of Immunology, Lab of Dendritic Cell Differentiation & Regulation, School of Medicine, Konkuk University, Chungju, 380-701 South Korea; 5 World Institute of Kimchi, Gwangju, 61755 Korea; 6 Division of Biotechnology, College of Environmental and Bioresource Sciences, Chonbuk National University, Iksan, 570-752 Korea

## Abstract

Activated macrophages have a greater ability of phagocytosis against pathogens that is mediated by large-scale actin rearrangement. However, molecular machineries that conduct this task have not been fully identified. Here, we demonstrate an unanticipated role of TAGLN2, a 22-kDa actin-binding protein, in Toll-like receptor (TLR)-stimulated phagocytosis. TAGLN2 was greatly induced in macrophages in response to lipopolysaccharide (LPS), a ligand for TLR4, partly via the NF-κB pathway. TAGLN2-deficient macrophages (*TAGLN2* ^−/−^) showed defective phagocytic functions of IgM- and IgG-coated sheep red blood cells as well as bacteria. Cell signaling pathways involved in actin rearrangement—PI3 kinase/AKT and Ras-ERK—were also down-regulated in LPS-stimulated TAGLN2-deficient macrophages. Moreover, *TAGLN2* ^−/−^ mice showed higher mortality after bacterial infection than wild-type littermates. Thus, our results revealed a novel function of TAGLN2 as a molecular armament required for host defense.

## Introduction

Bacterial infection is a common cause of sepsis and sepsis-associated organ failure, which cause high morbidity and mortality^1^. Despite modern advances in critical care, the pathogenesis of this devastating pathogen remains unclear. Macrophages are the first line of host defense against pathogens and phagocytosis is one of the most important initial immune responses. Patients with phagocytic defects typically experience early dissemination of infection, leading to severe sepsis and increased mortality^2^. Moreover, reduced phagocytic activity during the first 24 h after admission has been recognized as a negative predictor for survival in septic patients^3^.

Macrophages can be activated by cytokines such as IFN-γ or bacterial endotoxins, such as lipopolysaccharide (LPS)^4^ and activated macrophages have a greater ability of phagocytosis against complement- and immunoglobulin-opsonized pathogens. During clearance of pathogens, upon receptor-mediated binding of the target, PI3 kinases (PI3K) and AKT kinases (AKT) are activated and required for dynamic and rapid reorganization of the actin cytoskeleton^5–7^. The polymerization actin cytoskeleton mediates protrusions or ruffling of the macrophage membrane that can internalize target pathogens. In this process, a large number of actin-regulatory proteins, which are responsible for nucleation, severing or depolymerization, bundling, and crosslinking, are participating in actin remodeling^8^. However, the molecular machineries that account for the enhanced ability of phagocytosis in activated macrophages as compared to resting macrophages are relatively unknown.

Transgelin (TAGLN), a 22-kDa actin-binding protein, was first discovered in chicken gizzard smooth muscle (SM22) with unknown function^9^, and thereafter it was named as “transgelin” because of its transformation sensitive and rapid actin-gelling properties^10^. In addition to TAGLN1 (also known as SM22α), two of its isoforms—TAGLN2 (SM22β) and TAGLN3 (NP25)—were identified in humans with ~70% amino acid sequence homology. Both isoforms, similarly to TAGLN1, are associated with actin filaments, but differ in cell type expression specificity. TAGLN1 is best characterized as an early marker of smooth muscle differentiation^11, 12^. TAGLN1 ablation in mouse reduces vascular contraction in response to depolarization and is involved in the process of arterial diseases^13^. TAGLN1 is also considered as a tumor suppressor, as loss of its expression is an early event in cell transformation and the development of some tumors^14^. TAGLN3 participates in the brain developments and influences neurite outgrowth^15^. The level of TAGLN3 is connected with neuropathological changes in the brain of alcoholic and schizophrenics^16^. Recent proteomic reports demonstrate that TAGLN2 is upregulated in certain tumors including colorectal^17^, hepatocellular^18^, breast^19^, and lung cancer^20^, thereby suggesting a role in tumor progression or metastasis. As compared to the TAGLN1 and 3, however, function of TAGLN2 in normal cellular processes is relatively uncharacterized.

Recently, we reported that TAGLN2 is predominantly expressed in T cells and stabilizes cortical filamentous (F-)actin, thereby maintaining F-actin contents at the immunological synapse, which allows effector T cells to efficiently kill cancer cells^21^. Further, we discovered that TAGLN2 in B cells also mediates synapse stabilization^22^. In contrast to the T and B cells, however, macrophages either derived from peritoneal cavity or bone marrow express very low level of TAGLN2, suggesting its minimal function in resting macrophages. Surprisingly, however, we found that TAGLN2 is greatly induced by lipopolysaccharide (LPS)—a ligand for TLR4—which also augments actin polymerization. This fact led us to investigate whether TAGLN2 corresponds to the dynamic actin rearrangement, and thus membrane protrusion or ruffling in activated macrophages. Here, we demonstrated that TAGLN2 mediated large-scale actin dynamics during the engulfment of bacteria, thereby augmenting phagocytosis, and that the regulation of phagocytosis by TAGLN2 played a pivotal role in host defense against bacterial infection.

## Results

### TAGLN2 is over-induced in response to LPS in macrophages

**Figure 1:**
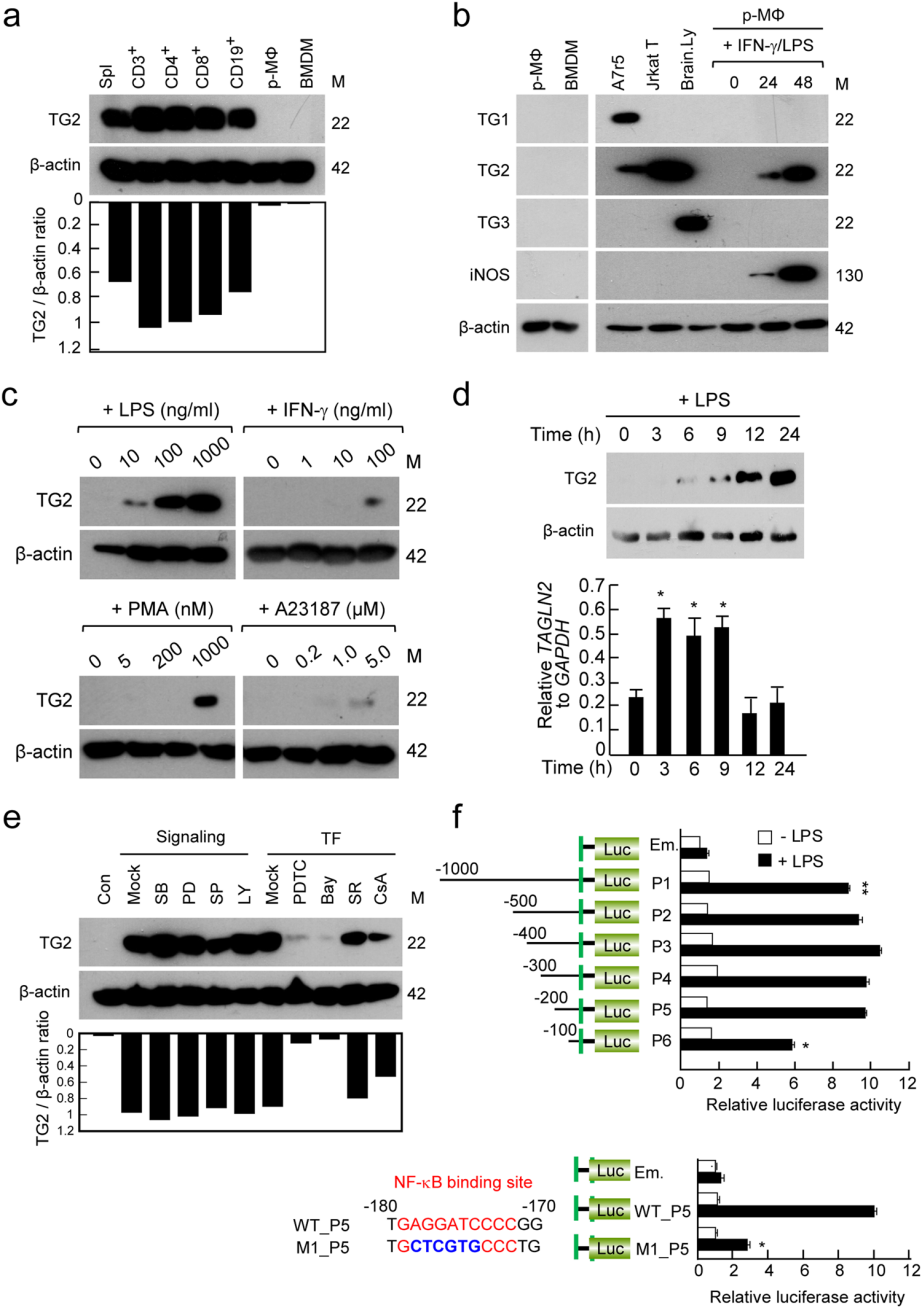
TAGLN2 is over-induced in response to LPS in macrophages. (**a**–**e**) Analysis of TAGLNs expression by western blot. (**a**) Expression of TAGLN2 in splenocytes (spl), T-cell subsets (CD3^+^, CD4^+^, and CD8^+^), a B-cell subset (CD19^+^), and macrophages (peritoneal, p-MΦ and bone marrow-derived, BMDM) purified from wild-type mice. TAGLN2 expression levels were quantitated as the ratio to β-actin by densitometry. Results are representative of three independent experiments. Full-length blots/gels are presented in Supplementary Fig. S5. (**b**) Expression of TAGLN1, 2, and 3 expression in peritoneal and bone marrow-derived macrophages. A7R5 cells, Jurkat T, and brain lysate were used as positive controls. Peritoneal macrophages were stimulated for indicated time with IFN-γ and LPS, and the expression of TAGLN1, 2, and 3, and iNOS (a positive control) was assessed. Full-length blots/gels are presented in Supplementary Fig. S5. (**c**) Peritoneal macrophages were incubated with various concentrations of LPS, IFN-γ, PMA (PKC activator), and A23187 (calcium activator) for 24 h. Full-length blots/gels are presented in Supplementary Fig. S6. (**d**) Peritoneal macrophages were treated with LPS (1 μg/mL) for different times as indicated. Western blot was performed as described in (**a**). Full-length blots/gels are presented in Supplementary Fig. S6. Expression of *TAGLN2* mRNA was quantitated by real-time quantitative PCR as compared to *GAPDH* mRNA expression. Data are shown as the mean relative expression ± SEM. *P < 0.05 *vs*. 0 time. (**e**) Peritoneal macrophages were pretreated for 30 min with various signaling modulators (SB, SP, PD, and LY (10 μM); SR (1 μM); CsA, PDTC, and Bay (10 μM)) and then the cells were further stimulated with LPS for 24 h. Full-length blots/gels are presented in Supplementary Fig. S7. (**f**) TAGLN2 promoter activity was analyzed by luciferase reporter assay using TAGLN2 promoter deletion or point-mutated constructs. All promoter fragments are indicated in the left side of the graph. The promoter activity was analyzed using peritoneal macrophages under indicated conditions.

Previously, we reported that TAGLN2 is highly expressed in immune-related tissues, such as thymus, spleen, and lymph nodes^21^. Moreover, it is constitutively expressed in primary T (CD3^+^, CD4^+^, and CD8^+^) and B (CD19^+^) cells (Fig. 1a). In contrast to the lymphoid cells, we noticed that macrophages derived from either bone marrow or peritoneum express minimum levels of TAGLN2 (Fig. 1a and Supplementary Fig. S1a and b), suggesting that it has a minimal function in resting macrophages. Surprisingly, macrophages treated with IFN-γ and LPS highly expressed TAGLN2 but no other TAGLN family members (Fig. 1b), suggesting a unique function of TAGLN2 after bacterial infection. Further study demonstrated that TAGLN2 expression was induced in response to LPS but not IFN-γ (Fig. 1c). In order to understand potential signals that mediate the induction of TAGLN2, two pharmacologic activators were used. Interestingly, TAGLN2 was significantly induced after treatment with phorbol 12-myristate 13-acetate (PMA), a protein kinase C (PKC) activator, and slightly induced after treatment with A23187, a calcium ionophore (Fig. 1c), suggesting that PKC, and partly calcium signaling, are likely involved in LPS-mediated TAGLN2 expression (Fig. 1c). Time-course analysis demonstrated that TAGLN2 mRNA induction by LPS preceded protein expression (Fig. 1d). We also used various pharmacologic inhibitors to determine the signaling pathway(s) that regulate(s) TAGLN2 expression. As shown in Fig. 1e, LPS-induced TAGLN2 expression was not changed after pretreatment with inhibitors of p38 kinase (SB203580), JNK (SP600125), ERK (PD098059), and PI3 kinase (LY294002) at the concentration that blocked the phosphorylation of the target proteins (data not shown). We next determined the transcription factors that were involved in the regulation of TAGLN2 expression. Whereas SR, an AP1 inhibitor, did not affect TAGLN2 expression, CsA, a NFAT pathway inhibitor, slightly decreased TAGLN2 expression in response to LPS (Fig. 1e). This result might reflect the slight induction of TALGN2 expression in response to the calcium ionophore, A23187 (Fig. 1c). Interestingly, the induction of TAGLN2 expression by LPS was almost completely suppressed after treatment with NF-κB inhibitors, PDTC and Bay11-7082 (Fig. 1e). These results strongly suggested that TAGLN2 expression was influenced by the NF-κB pathway. We therefore analyzed the upstream promoter region of three TAGLN family members and interestingly found that TAGLN2 only contains NF-κB consensus motif at the −174 ~  −179 upstream region (see Supplementary Fig. S2). Consistently, point mutation of this region significantly reduced the NF-κB luciferase activity (Fig. 1f).

### Defective phagocytosis in macrophages from *TAGLN2*^−/−^ mice

**Figure 2:**
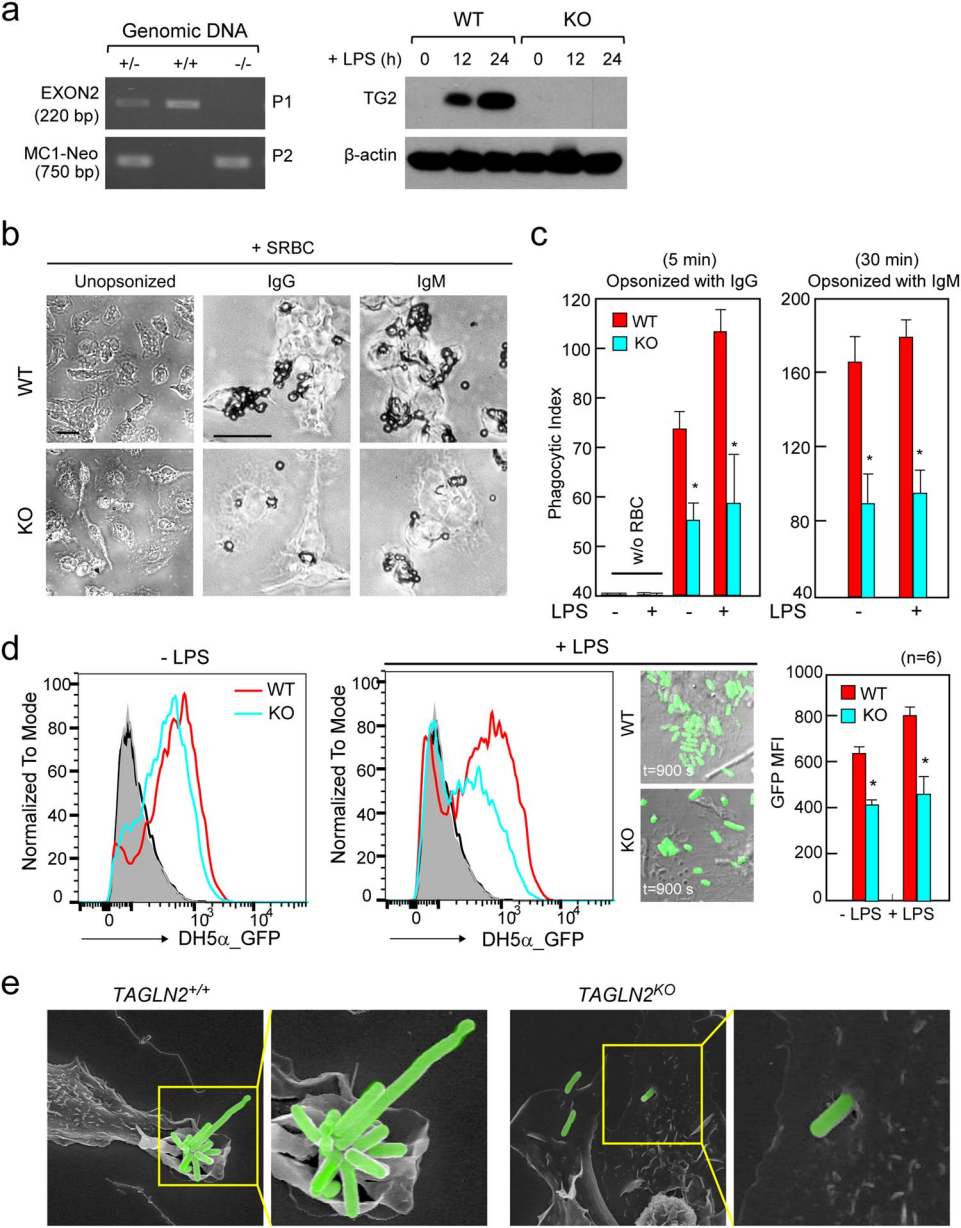
Defective phagocytosis in macrophages from *TAGLN2* ^−/−^ mice. (**a**) PCR genotyping. Genomic DNA was extracted from mouse ear tissue. The wild-type (+/+) and heterozygote mutant (+/−) alleles were detected with primer (P1) targeting EXON2, whereas the homozygote (−/−) alleles were not detected with this primer. Using the P2 (MC1-Neo) primer set, the heterozygote and homozygote alleles, but not the wild-type allele, were detected. TAGLN2 protein in macrophages from wild-type and *TAGLN2* ^−/−^ mice was determined by Western blot analysis. Isolated cells were stimulated with LPS (1 μg/mL) for 12–24 h. Results are representative of three independent experiments. (**b** and **c**) Impaired FcγR- or CR-mediated phagocytosis in *TAGLN2* ^−/−^ macrophages. Full-length blots/gels are presented in Supplementary Fig. S8. (**b**) Peritoneal macrophages from wild-type and *TAGLN2* ^−/−^ mice were activated with LPS (1 μg/mL) for 24 h, incubated with IgG- or IgM-opsonized sRBC, fixed, and imaged at various time points (IgG, 5 min; IgM, 30 min). Scale bars, 5 μm. (**c**) The phagocytic index was calculated as the number of internalized sRBC in 100 macrophages. *P < 0.05 *vs*. wild-type macrophages. (**d**) Phagocytic activity *in vivo*. GFP-expressing *E. coli* was injected into the peritoneum of recipient wild-type and *TAGLN2* ^−/−^ mice. The phagocytic function was calculated by flow cytometry as the percentage of GFP-positive in F4/80-APC-gated cells. *P < 0.05, *n* = 10. Phagocytosis of GFP-expressing *E. coli* in wild-type and *TAGLN2* ^−/−^ macrophages stimulated with or without LPS for 24 h. Time-lapse imaging of *in vitro* phagocytosis of GFP-expressing *E. coli* was performed with confocal microscopy (FV1000). Selected last still images from time-lapse movies (see Supplementary Videos S3–S4) are shown (t = 900 s). The imaging was started after the addition of GFP-expressing *E. coli*. Scale bars, 5 μm. Quantification of phagocytosed *E. coli* cells by macrophages was represented as bar graph. *P < 0.05, *n* = 6. (**e**) Macrophages from (**d**) were exposed to the *E. coli* for 10 min, fixed, and processed for SEM. Scale bars, 5 μm. Green fluorescence in the figure was pseudo-colored.

A possible association between TLR signaling and phagocytosis has been suggested previously^23^. In addition, TLR signaling events are linked with the actin cytoskeleton dynamics^24^. As TAGLN2 is an actin-binding protein and has been reported to regulate actin dynamics^21, 25^, we first examined whether function of TAGLN2 is related with the receptor-mediated phagocytosis. To this end, we utilized macrophages obtained from TAGLN2-deficient (*TAGLN2* ^−/−^) mice^21^. *TAGLN2* gene disruption and protein deficiency were confirmed by genotyping and western blotting (Fig. 2a). Peritoneal macrophages from *TAGLN2* ^+/+^ and *TAGLN2* ^−/−^ mice were activated with LPS for 24 h and then challenged with IgG- or IgM-opsonized sRBCs. *TAGLN2* ^−/−^ macrophages phagocytosed fewer sRBCs than *TAGLN2* ^+/+^ macrophages in both FcγR- and complement receptor (CR)-mediated pathways (Fig. 2b). Quantitative analysis of phagocytosed sRBCs on the basis of phagocytic index is shown in Fig. 2c. A process of engulfment by micropinocytosis also requires actin cytoskeleton reorganization. We then determined the involvement of TAGLN2 in macropinocytosis using dextran. The uptake of 70-kDa dextran by resting microphages in *TAGLN2* ^−/−^ mice was significantly lower than that in their wild-type counterparts (see Supplementary Fig. S3). As reported previously^31^, however, macropinocytosis was down-regulated in macrophages treated with LPS, and therefore no significant difference was observed in the number of macropinosomes per cell.

We next assessed phagocytosis of live bacteria *ex vivo* and *in vivo*. To this end, peritoneal macrophages (*TAGLN2* ^+/+^ and *TAGLN2* ^−/−^) were treated with or without LPS and then incubated with GFP-transfected live bacteria. *TAGLN2* ^−/−^ macrophages showed attenuated phagocytosis (Fig. 2d and Supplementary Videos S1–S4). The slightly reduced phagocytic activity in resting *TAGLN2* ^−/−^ macrophages suggested that a trace level of TAGLN2 maintains phagocytic function (Fig. 2d). SEM analysis revealed that LPS-stimulated wild-type macrophages efficiently captured the infecting bacteria in large membrane ruffles, whereas *TAGLN2* ^−/−^ macrophages captured relatively low numbers of bacteria in small ruffles (Fig. 2e).

### TAGLN2 regulates actin polymerization in activated macrophages

**Figure 3:**
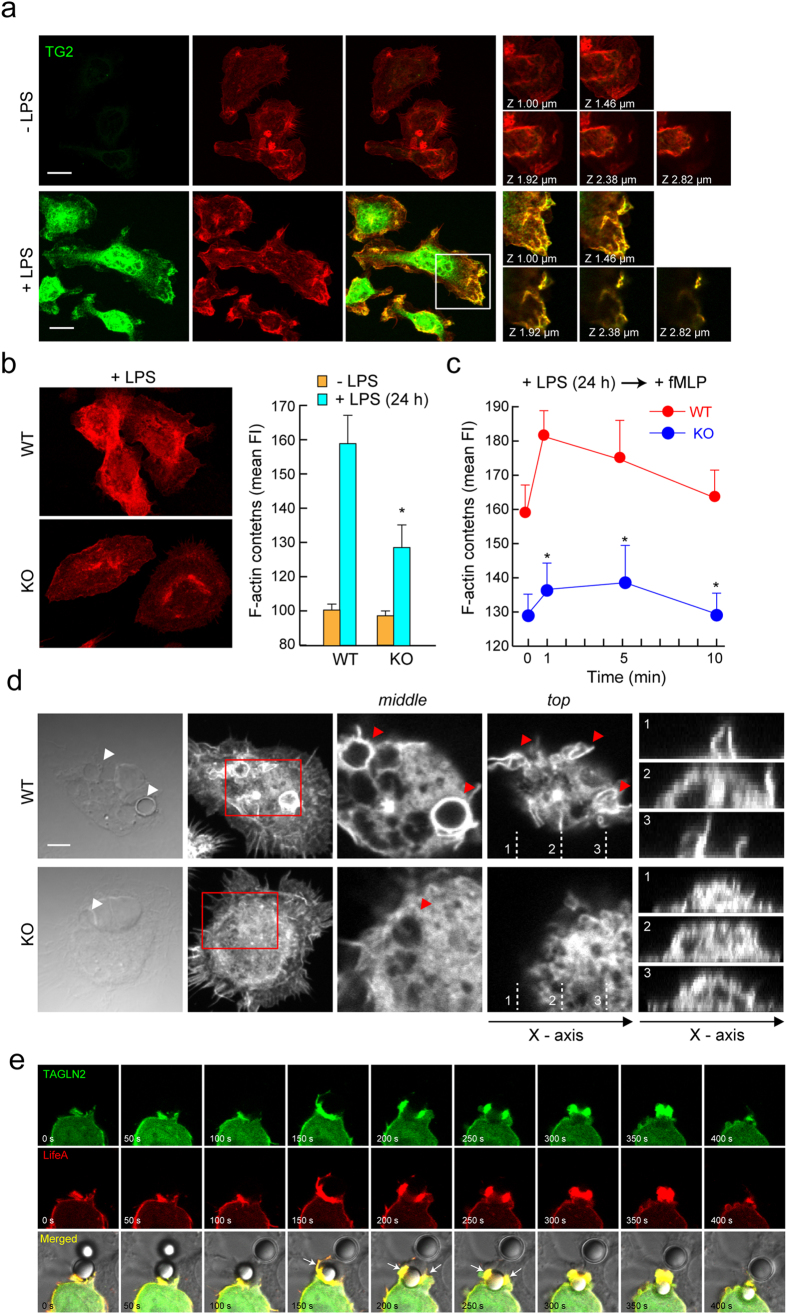
Impaired actin polymerization in TAGLN2-deficient macrophages. (**a**) TAGLN2 is localized at the actin-rich membrane ruffles. Freshly isolated peritoneal macrophages were incubated with or without LPS (1 μg/mL) for 24 h, and were serum-starved for 3 h. Cells were treated with fMLP (200 nM) for 2 min, fixed, stained with anti-TAGLN2 antibody and TRITC-phalloidin, and observed by confocal microscopy. Scale bars, 10 μm. Single z-slices were shown in basal and dorsal views where TAGLN2 and F-actin colocalize in ruffles. The boxed areas (white) are represented as zoomed images in the right micrographs. (**b**) WT or *TAGLN2* ^−/−^ macrophages were stimulated with LPS for 24 h and analyzed for their actin contents and polymerization by confocal microscopy (left) flow cytometer (right) after stained with TRITC-phalloidin. Data are represented as the mean value of fluorescent intensity (FI). (**c**) WT or *TAGLN2* ^−/−^ macrophages were pretreated with LPS for 24 h and then stimulated with fMLP for the indicated time periods. Actin polymerization was analyzed as in Fig. c. (**d**) Impaired actin accumulation at phagocytic cups in *TAGLN2* ^−/−^ macrophages. Middle and top panels show F-actin enrichment at phagocytic cups and dynamic ruffle formation during phagocytosis, respectively. Arrows point to phagocytic cups surrounding sRBC. Scale bars, 5 μm. (**e**) RAW 264.7 cells were transfected with TAGLN2_GFP and LifeAct. At 24 h after transfection, cells were allowed to phagocytose IgG-coated beads for 5 min. Images were captured as a T-series at every 20 s for 10 min. Actin (red) and TAGLN2 (green) were localized on the phagosome and pseudopodia at the very early stages with similar kinetics. Selected last still images from time-lapse movies (see Supplementary Video S5) are shown.

To understand how TAGLN2 regulates phagocytosis in macrophages, properties of TAGLN2 in relation to actin dynamics were studied. Primary macrophages pretreated with LPS were fixed, and stained for TAGLN2 and F-actin. We found that TAGLN2 intensively accumulated at the membrane F-actin-rich apical region of cells (Fig. 3a). To examine whether TAGLN2 affects actin polymerization, TAGLN2-deficient macrophages were stimulated with LPS and then stained for F-actin. As shown in Fig. 3b, LPS-induced actin polymerization was significantly reduced in TAGLN2-deficient (*TAGLN2* ^−/−^) primary macrophages. We next examined whether accumulated TAGLN2 also affects the fMLP-induced transient actin polymerization. Wild-type macrophages pretreated with LPS exhibited stronger actin polymerization in response to the fMLP than TAGLN-2-deficient macrophages (Fig. 3c), suggesting that TAGLN2 plays an important role for the actin cytoskeleton dynamics.

The initial step of phagocytosis is believed to form phagocytic cups through a process that involves actin polymerization under the plasma membrane. To this ends, individual macrophages from *TAGLN2* ^+/+^ and *TAGLN2* ^−/−^ mice were incubated with IgG-opsonized sRBCs, and then the patterns of F-actin were observed. The F-actin-rich “cup”-like structures were clearly seen in wild-type macrophages. However, much less accumulating F-actin signals at the contact region of sRBC were found in *TAGLN2* ^−/−^ macrophages (Fig. 3d). These results suggest that TAGLN2 plays a crucial role for actin reorganization in LPS-activated macrophages. To observe actin assembly and the role of TAGLN2 during phagocytosis, we had to visualize both actin and TAGLN2, simultaneously and live. To this end, RAW 264.7 cells were co-transfected with GFP-tagged TAGLN2 (TAGLN2_GFP) and mCherry-tagged LifeAct (LifeAct). In response to opsonized IgG beads, TAGLN2 was highly enriched around the phagocytic cup along with LifeAct signals (Fig. 3e and Supplementary Video 5). This data provided further evidence that TAGLN2 contributed to phagocytosis through actin regulation.

### TAGLN2 mediates membrane ruffling in LPS-stimulated macrophages

**Figure 4:**
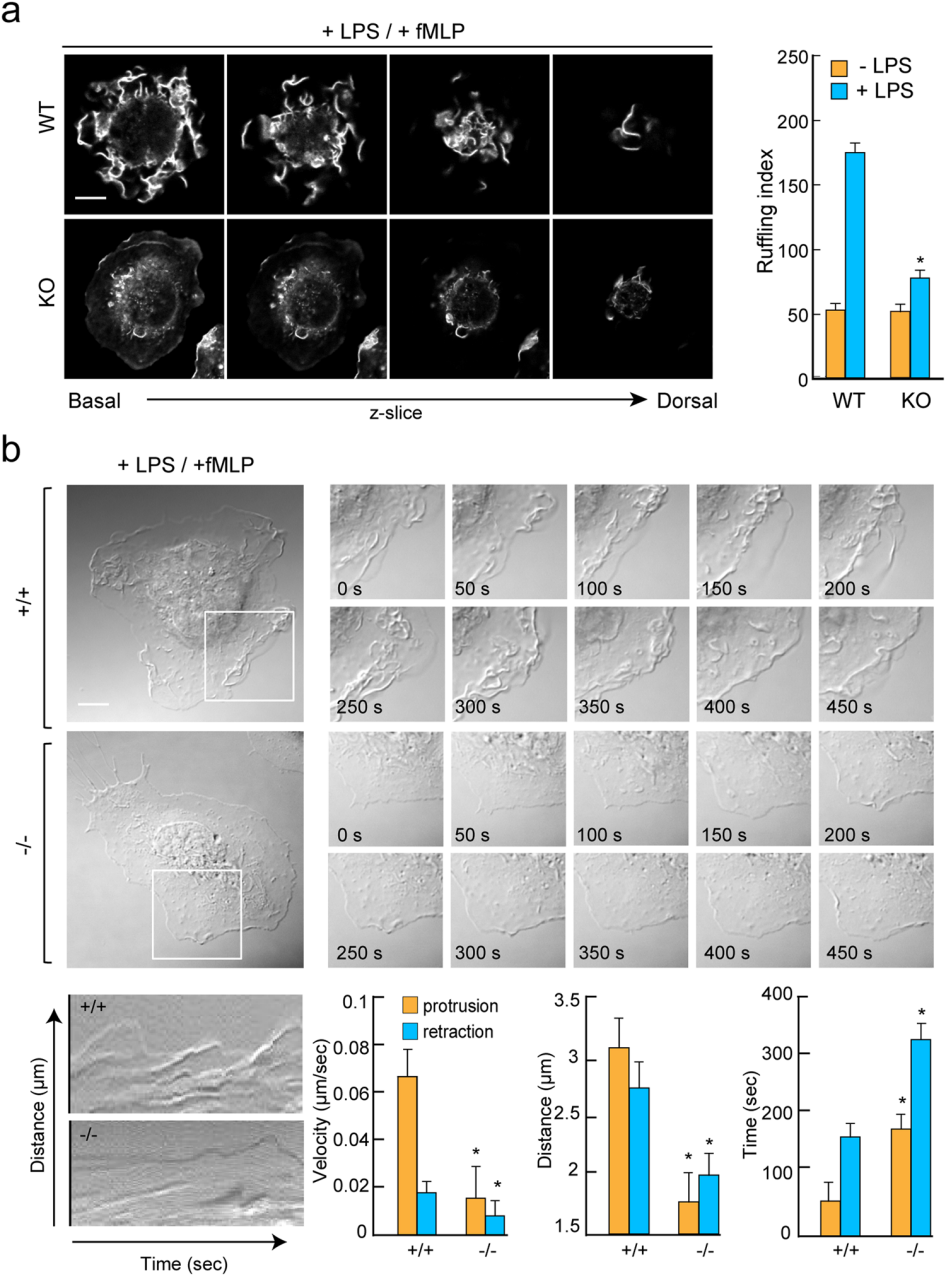
TAGLN2 mediates membrane ruffling in LPS-stimulated macrophages. (**a**) Membrane ruffling formation in fMLP (200 nM)-stimulated peritoneal macrophages from wild-type and *TAGLN2* ^−/−^ mice. LPS-stimulated and serum-starved peritoneal macrophages were treated with fMLP for 2 min, fixed, and stained for F-actin with TRITC phalloidin. Scale bars, 5 μm. Quantification of fMLP-induced membrane ruffles in wild-type and *TAGLN2* ^−/−^ macrophages by ruffling index. Data are expressed as means ± SEM of the ruffling index of four random fields. *P < 0.05 *vs* LPS-treated wild-type macrophages. This result is a representative of three independent experiments. (**b**) Lack of membrane protrusive activity in *TAGLN2* ^−/−^ macrophages. Representative frames from time-lapse videos and kymographs obtained after fMLP stimulation. Peritoneal macrophages from wild-type and *TAGLN2* ^−/−^ mice were serum-starved for 3 h before fMLP stimulation. DIC images were collected at 10-second intervals for 10 min (see Supplementary Videos S6–S7). Multiple kymographs (*n* = 4–6) were generated from each cell using the EMBL ImageJ software. Quantitative analysis of protrusion activity (velocity, distance, and time) included a total of three different regions per treatment from each experiment (*n* = 3 independent experiments). Scale bars, 5 μm. (**c**) SEM images of ruffling in wild-type and *TAGLN2* ^−/−^ macrophages. Scale bars, 5 μm.

We next investigated whether actin dynamics by TAGLN2 is connected with the formation of membrane ruffle—an important cellular structure for phagocytosis or macropinocytosis of foreign substances. Wild-type (*TAGLN2* ^+/+^) macrophages showed multiple F-actin-positive membrane ruffles after stimulation with LPS/fMLP (Fig. 4a), whereas *TAGLN2* ^−/−^ macrophages presented limited, localized patches of ruffling with much lower phalloidin-TRITC intensity (Fig. 4a). Flow cytometric analysis of surface markers demonstrated that TAGLN2-deficiency had no effect on macrophage differentiation (see Supplementary Fig. S4). Wild-type macrophages exhibited cycles of lamellipodial protrusion and retraction and phase-dark membrane ruffles, yielding kymographs that looked like “rolling hills” (Fig. 4b and Supplementary Video S6–S7). In contrast, *TAGLN2* ^−/−^ macrophages exhibited lamellipodial protrusion, retraction, and phase-dense membrane ruffling at a significantly lower frequency, yielding more featureless “prairie-like” kymographs (Fig. 4b). Computer-assisted kymograph and line-scan analyses revealed that the mean velocity and distance of membrane protrusion and retraction are significantly decreased in *TAGLN2* ^−/−^ macrophages (Fig. 4b). Similar to areas of phagocytic cup formation (Fig. 3e), TAGLN2 was also completely co-localized with F-actin (LifeAct) in areas with membrane ruffling and lamellipodia extension—sites of enhanced actin polymerization-depolymerization—in RAW264.7 cells treated with PMA (Supplementary Video 8).

### TAGLN2 enhances phagocytosis through PI3K/AKT and ERK signaling pathways

**Figure 5:**
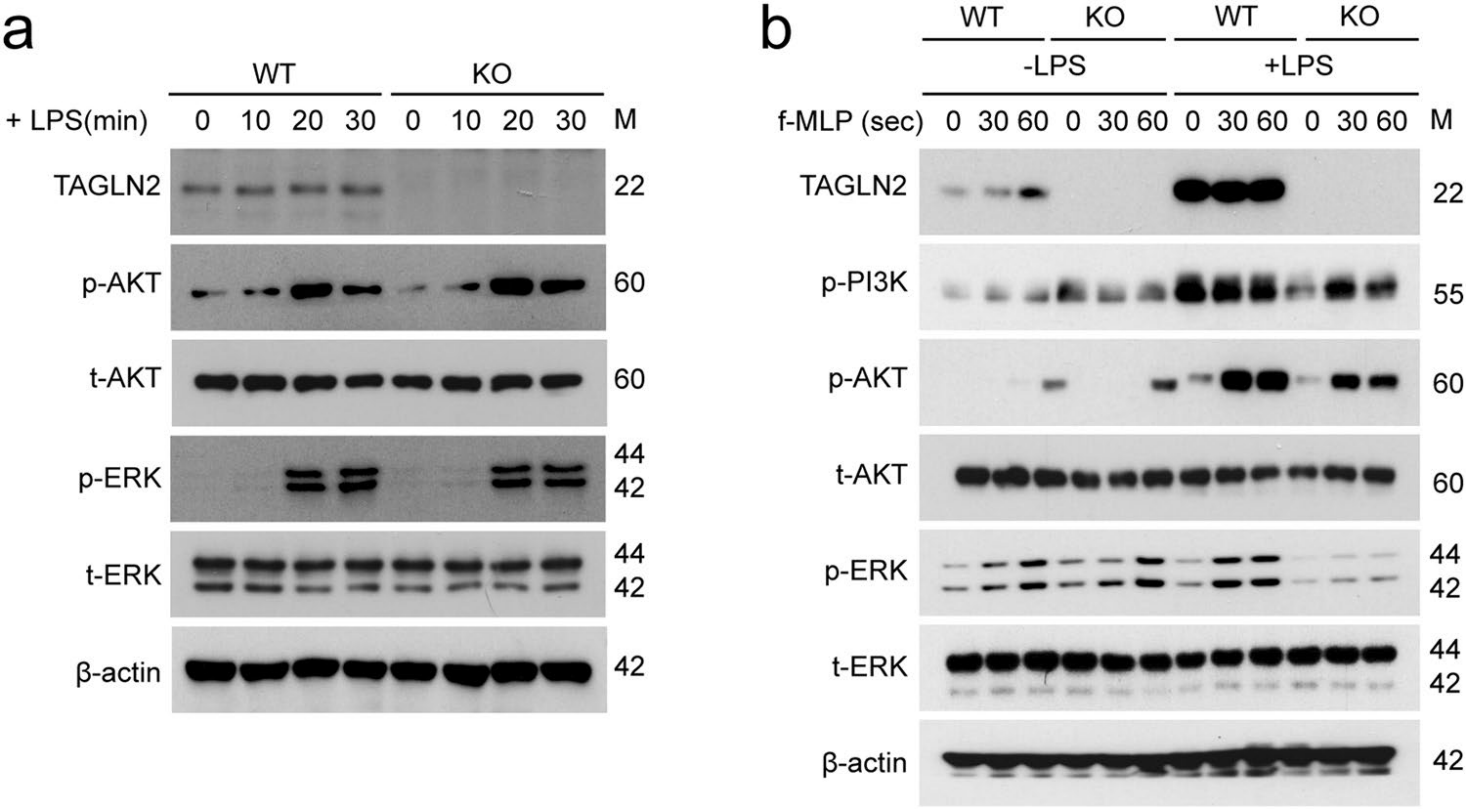
TAGLN2 is required for the activation of PI3 kinase/AKT and Ras-ERK signaling pathways. (**a**) Peritoneal macrophages from wild-type and *TAGLN2* ^−/−^ mice were stimulated with LPS (1 μg/mL) for the indicated time points. The phosphorylated and total forms of AKT, ERK, and JNK were detected by western blot. (**b**) Peritoneal macrophages were pre-activated with LPS (1 μg/mL) for 24 h. Cells were serum-starved for 3 h and stimulated with fMLP (1 μM) for the times indicated. Cell lysates were subjected to western blot with anti-phospho and total PI3K, AKT, ERK, and JNK. The data are representative of three independent experiments. Full-length blots/gels are presented in Supplementary Fig. S9.

It is well established that polymerization of F-actin at initial sites of phagocytosis is dependent on the PI3 kinase (PI3K)/AKT signaling cascades^5^. In addition, the Ras-MAPK signal transduction pathway and the ERK-related pathways, which are involved in signal transduction from receptor tyrosine kinases, growth factor receptors, some G-protein-coupled receptors, and TLRs, are also known to promote the proliferation, differentiation, and particularly the activation and polarization of macrophages^26^. We therefore tested whether TAGLN2 is connected with PI3K/AKT and MAPK signaling pathways in macrophages. We first investigated these signaling events in resting macrophages in response to the LPS—a TLR4-mediated signaling. Interestingly, TAGLN-deficiency had little effect on LPS-mediated activation of AKT and MAPKs including ERK and JNK (Fig. 5a), suggesting that TAGLN2 is not directly involved in TLR4-mediated activation of Ras-ERK or PI3K signaling pathways. As a second evaluation, we measured the fMLP-induced signaling events in resting or LPS-activated macrophages (for 24 h). Surprisingly, fMLP-mediated activations of PI3K/AKT as well as ERK were not different in resting condition of both macrophages (Fig. 5b). However, these events were significantly down-regulated in TAGLN2-knockout macrophages activated by LPS (Fig. 5b). These results strongly suggest that TAGLN2 is connected with the actin polymerization and rearrangement during bacterial phagocytosis.

### *TAGLN2*^−/−^ mice show enhanced susceptibility to bacterial peritonitis

**Figure 6:**
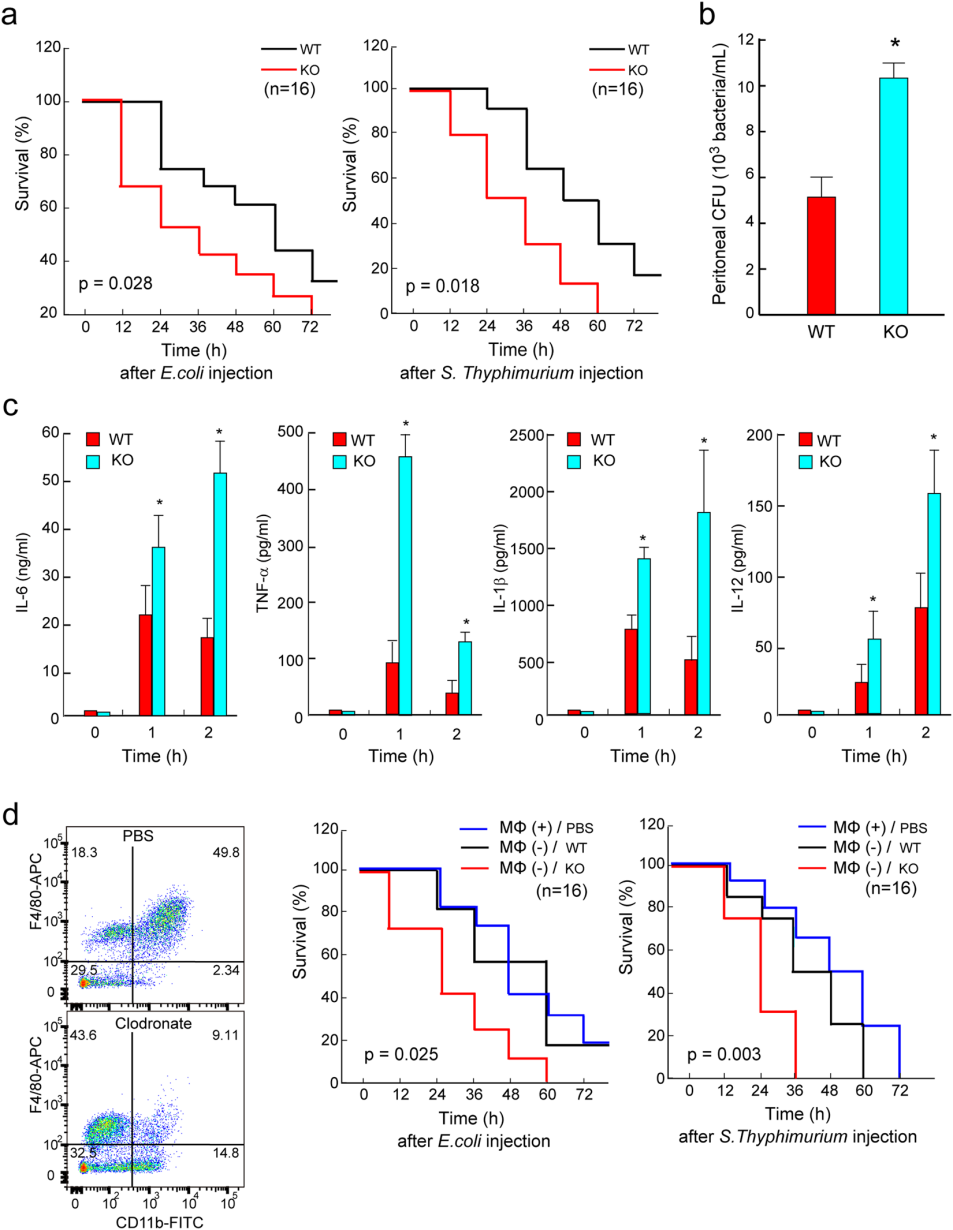
TAGLN2 participates in host survival after bacterial peritonitis. (**a**) *TAGLN2* ^−/−^ mice demonstrate reduced survival after bacterial peritoneal sepsis. Age-, sex-, and weight-matched wild-type (black line) and *TAGLN2* ^−/−^ (red line) mice were injected i.p. with 5 × 10^8^ CFU of live *E. coli* (DH5α) or 5 × 10^6^ CFU of live *S. typhimurium*. Kaplan-Meier survival curves revealing the proportion of mice that are alive in each group as a function of time from the first injection of bacteria are shown. (**b**) The *E. coli* cell count in peritoneal lavage fluid from wild-type or *TAGLN2* ^−/−^ mice 4 h after i.p. administration of 10^8^ CFU/mouse of *E. coli* is shown as bar graph (*n* = 6). *P < 0.05 *vs* LPS-treated wild-type macrophages. (**c**) Cytokine (TNF-α, IL-1β, IL-6, and IL-12) levels in plasma at 0, 1, and 2 h after i.p. injection of 5 × 10^6^ CFU of live *S. typhimurium* into wild-type and *TAGLN2* ^−/−^ mice. Data are the mean ± SEM; *n* = 8 per group. *i < 0.05 *vs*. wild-type mice at the same time point. (**d**) Flow cytometry plots of CD11b^+^, F4/80^+^ macrophages isolated from spleens at day 2 after clodronate liposomes treatment for depletion (n = 3 per group). Survival after transfer of wild-type and *TAGLN2* ^−/−^ peritoneal macrophages in recipient wild-type mice with *E. coli-* or *S. typhimurium*-induced sepsis (*n* = 15/group).

Finally, we determined whether regulation of phagocytosis by TAGLN2 affects the host response to bacterial infection. Intraperitoneal inoculation of GFP-expressing *Escherichia coli* resulted in a mortality of ~50% after 48 h in wild-type mice (Fig. 6a). The Kaplan-Meier survival curves for wild-type and *TAGLN2* ^−/−^ mice injected with *E. coli* and *Salmonella* revealed that *TAGLN2* ^−/−^ mice showed significantly (*p* = 0.028 and 0.018, respectively) increased mortality as compared to their wild-type counterparts on each day after injection. For *Salmonella* infection, similar results were obtained (Fig. 6a). At 4 h postinfection with 10^8^ CFU of E. coli, *TAGLN2* ^−/−^ mice displayed more (~2-fold) bacteria in the peritoneal lavage fluid than did WT mice (Fig. 6b), indicating defective bacterial clearance in the *TAGLN2* ^−/−^ mice. Sepsis is characterized by inefficient bacterial clearance and the overexpression of inflammatory cytokines^1^. Therefore, we examined the effects of TAGLN2 knockout on cytokine production during *Salmonella*-induced sepsis. Notably, 1–2 h after infection, *TAGLN2* ^−/−^ mice showed higher plasma levels of TNF-α, IL-6, IL-1β, and IL-12 than wild-type mice (Fig. 6c). Finally, as the *TAGLN2* ^−/−^ mice used in this study were whole-body knockout mice, it was impossible to discriminate between primary and secondary effects of TAGLN2 deficiency in other tissues or cells. Therefore, we performed adoptive transfer of peritoneal macrophages following pharmacological depletion of macrophages in a mouse model of bacterial sepsis. Mice with adoptively transferred *TAGLN2* ^−/−^ macrophages showed higher mortality in response to both DH5α and *Salmonella* than wild-type macrophages (Fig. 6d). These results demonstrated that TAGLN2 is a corresponding factor for macrophage-mediated phagocytic function against bacterial infection, thus ameliorating bacterial sepsis.

## Disscusion

To effectively defeat pathogens, macrophages exhibit advanced phagocytic activity^27, 28^. However, factors of membrane protrusion or ruffling, which is intricately connected to the phagocytic functions and occurs after contact with bacterial pathogens, are not fully understood. Originally, TAGLN1 was identified as actin-gelling or actin-crosslinking protein and became accepted as a marker of smooth muscle differentiation^10, 11^. TAGLN3 (=NP25) was discovered later and known to function in neurite outgrowth^15^. However, function of TAGLN2 was relatively unknown. In this report, we have shown that TAGLN2 induction by LPS plays a pivotal role in the phagocytic function of macrophages. This is conclusively demonstrated by the reduced phagocytic functions of IgG- or IgM-coated sRBC and *E. coli* by TAGLN2-deficient macrophages stimulated with LPS. Actin polymerization and PI3K/AKT and ERK activation were also significantly impaired in TAGLN2-deficient macrophages. Notably, mice with *TAGLN2* ^−/−^ macrophages showed higher mortality after bacterial peritonitis. Thus, our findings suggest that TAGLN2 is an essential factor of macrophages for host defense, maintaining protective immunity in bacterial sepsis.

The fact that TAGLN2 is over-induced in response to LPS in both peritoneal and bone marrow-derived macrophages definitely suggests that it plays a role in macrophages in relation to the infection. However, resting macrophages have preexisting mechanisms for internalizing and clearing microbial materials through receptor-mediated phagocytosis. Thus, the question arises is why macrophages need additional molecule like TAGLN2 to engulf pathogens? Under certain conditions, receptor-mediated phagocytosis itself may not be sufficient for effective elimination of microbial pathogens. For example, some bacteria can modulate the macrophage killing capacity by disturbing receptor-mediated phagocytic function^29^. Thus, as an alternative to receptor-mediated phagocytosis, macrophages can also internalize materials via macropinocytosis which allows the internalization of large amounts of materials without any receptor engagement^30^. Indeed, we found that resting *TAGLN2* ^−/−^ macrophages had a reduced capacity to engulf dextran (TxR-dextran), indicating impaired macropinocytosis. However, macropinocytosis is known to be down-regulated in macrophages or dendritic cells treated with LPS, presumably to maximize the presentation of captured antigens^31^. We also observed that LPS-treatment significantly reduced macropinocytosis in both wild-type and TAGLN2-deficient macrophages. Therefore, it is unlikely that TAGLN2 expression is directly linked to macropinocytosis. However, the reduced capacity for macropinocytosis in TAGLN2-deficient macrophages indicated that TAGLN2 was also involved in macropinocytosis. Thus, it is possible that the presence of TAGLN2 broadly allows for the extension of the membrane surface, which establishes efficient particle contact and allows the binding of a higher number of bacteria per cell.

It was interesting to note that, among three TAGLN family members, TAGLN2 was the only isoform that can be induced by LPS stimulation. The inhibition of TAGLN2 expression by NF-κB inhibitors suggested a major role for NF-κB in the induction of TAGLN2 in macrophages. Consistent with this, the 5′ promoter region (−150 to −200 bp) of *TAGLN2* contains the binding sequence for NF-κB while other TAGLN family members do not have this sequence. Interestingly, we also found that TAGLN2 is induced by TNF-α, which is a triggering signal for macrophage activation, thereby suggesting that induction of TAGLN2 is controlled by the secondary triggering signals instead of priming signals like IFN-γ^32^.

The signaling upstream of TAGLN2 was not investigated in the present study. A previous report demonstrated that protein kinase C (PKC) acts upstream of TAGLN1 in vascular smooth muscle cells^33^. Moreover, PFTK, a cdc2-related serine/threonine protein kinase, controls TAGLN2 phosphorylation at S83 and S163 in hepatocellular carcinoma^34^. Both reports demonstrated that phosphorylation induces dissociation of TAGLNs from the actin cytoskeleton *in vivo* ^33, 34^, which is in consistence with the results of an *in vitro* study in which the substitution S181D, which mimics the phosphorylated form, reduced the binding of TAGLN1 to actin^13^. However, a more elaborate approach will be necessary to uncover the relevance of TAGLN2 phosphorylation *in vivo*. For instance, Lv *et al*. demonstrated that TAGLN1 phosphorylation induces actin depolymerization, whereas Leung *et al*. suggested that dephosphorylated TAGLN2 is an active form mediating depolymerization^34, 35^. Apart from the upstream signaling, however, we found that TAGLN2 influences the fMLP-mediated actin dynamics through the activations of PI3K/AKT signaling and Ras-ERK in LPS-activated macrophages^5^. These results suggested that TAGLN2 played an essential role in phagocytosis in activated macrophages, mainly through actin rearrangement at the site of bacterial contact.

Although we understand that TAGLN2 is an actin-binding protein that functions to bundle and stabilize actin filaments and also counteracts the cofilin action^21^, the main question is how this protein eventually can control membrane ruffling of activated macrophages. Interestingly, we recently found that TAGLN2 can polymerize G-actin under low salt condition where actin polymerization is normally completely suppressed (unpublished result). In fact, to our knowledge, only a few proteins can induce actin polymerization under low-salt conditions. Fragments of the skeletal muscle proteins nebulin and myosin S-1, as well as the vinculin tail region are known to bind actin and induce polymerization in low-salt conditions^36–38^. Both fesselin (avian smooth muscle) and caldesmon bind to G-actin and induce its transformation into F-actin in a variety of conditions including the virtual absence of salt^39, 40^. However, there are no indications that these fragments directly participate in membrane ruffling in the cells. Interestingly, however, it has been demonstrated LL-37, an antimicrobial peptide secreted from macrophages, polymorphonuclear leukocytes, and keratinocytes, induces G-actin polymerization—presumably via non-specific electrostatic interactions between LL-37 and actin under low salt condition^41^—and increases macrophage phagocytosis^42^. In addition, knockout of an LL-37 analog in mice (*Cnlp*−/−) diminished bacterial phagocytosis^42^. Therefore, to understand the mechanism of LL-37-mediated increased phagocytic activity, it would be worthwhile to investigate whether LL-37 controls macrophage membrane ruffling during phagocytosis. In addition to the LL-37, previous reports demonstrated that *Salmonella* invasion protein A (SipA) protein, which is one of the components of bacterially encoded type III protein secretion system (TTSS)−1^43, 44^, also extensively polymerizes G-actin *in vitro* and triggers large-scale membrane protrusions and ruffles at the site of *Salmonella* entry^43, 45^. If this is true, it is striking because this suggests that although both invasive pathogens and host macrophages have completely opposite purposes for phagocytosis, they use a highly similar mechanism to accomplish their goals.

Bacterial infection is a leading cause of sepsis which raises high morbidity and mortality^1^. Phagocytosis of bacteria is one of the most important initial immune responses that can block early dissemination of infected bacteria^2^. In this respect, development of cell-permeable peptides that exhibit TAGLN2 functions may have potential clinical value for the treatment of bacterial sepsis. The effects of cell-permeable TAGLN2 peptides in both macrophages and T cells are currently under the investigation.

In summary, we found that TAGLN2 is the only TAGLN family member which can be over-induced in response to the LPS in macrophages. After induction, TAGLN2 recruited to the membrane and induced large-scale cytoskeletal rearrangements that are necessary for membrane protrusion and ruffling, and eventually phagocytic function. These results strongly suggest that TAGLN2 is an important endogenic factor protecting the host from bacterial infection-related sepsis via controlling actin dynamics at the membrane ruffles. This study not only revealed an unexpected role for TAGLN2, but also offered important insights into the complex molecular armamentarium required for normal host defense.

## Materials and Methods

### Mice

C57BL/6 wild-type mice were purchased from Damul Science (Korea) and all mice were housed under specific pathogen-free conditions. TAGLN2 (*TAGLN2* ^−/−^) knockout mice have been described previously^21^. All experimental methods and protocols were approved by the Institutional Animal Care and Use Committee of the School of Life Sciences, Gwangju Institute of Science and Technology and carried out in accordance with their approved guidelines (IACUC GIST-2015-04).

### Cells

Jurkat T-cells (TIB-152), A7r5 (CRL-1444) cells, and RAW 264.7 cells (TIB71™) were maintained in RPMI-1640 or DMEM (Invitrogen) supplemented with 10% (v/v) FBS (Invitrogen). Mouse CD3^+^ T-cells were purified from the mouse spleen and lymph nodes on a T-cell enrichment column (R&D Systems). Mouse splenocytes were dispersed and purified into CD4^+^, CD8^+^, and CD19^+^ populations by MACS cell separation (Miltenyi Biotec, Bergisch Gladbach, Germany). The purity of each population was confirmed as >95% by flow cytometry. Mouse brain lysate was obtained from C57BL/6 wild-type mice. Peritoneal macrophage isolations were performed 60 h after an intraperitoneal injection of 2.5 mL 3% thioglycollate solution (Sigma) by peritoneal lavage with 5 mL of PBS. Withdrawn cells were cultured for 1 h at 37 °C and then rinsed to remove suspended cells. Of these adherent cells, >99% were F4/80^+^ macrophages. To prepare conditioned media for differentiation of BMDMs, L929 cells were cultured in DMEM with 10% FBS and 1% penicillin/streptomycin at 37 °C in a 5% CO_2_ incubator. Cell culture media were collected and filtered using 0.22-μm filters and kept at −20 °C. Mouse bone marrow cells were isolated from femur and tibia and cultured in RPMI-1640 supplemented with 30% of L929 cell-conditioned medium. After 6 days, BMDMs were confirmed to be >90% by flow cytometry with CD11b^+^ and F4/80^+^.

### cDNA constructs

The pEGFP-N1 (CMV promoter; Clontech, Mountain View, CA) vector encoding TAGLN2 (TG2) and LifeA_RFP were described in our previous paper^21^. Transcription factor binding sites of TAGLN1, 2, and 3 were identified using the computer program Factor (HUSAR program package, DKFZ Heidelberg). For promoter assay, human TAGLN2 promoter (1 kb upstream) was cloned by PCR using human CD3 T cell genomic DNA as a template. Mutants of the TAGLN2 promoter region which were indicated in the Fig. 1f were generated and verified by sequencing (Macrogen, Seoul, Korea). All obtained PCR products were cloned in front of the luciferase vecter pGL-2 (Promega, Heidelberg, Germany).

### Scanning electron microscopy

For scanning electron microscopy (SEM), thioglycollate-elicited peritoneal macrophages grown on Si-wafer were serum-starved for 3 h. Where indicated, cells were prestimulated with 1 μg/mL of LPS. The cells were then incubated with *E. coli* for 10 min or stimulated with fMLP for 2 min. After incubation, the cells were rinsed with PBS and processed for SEM.

### Confocal microscopy

For imaging of endogenous TAGLN2 localization, peritoneal macrophages were permeabilized with PBS-T for 10 min, blocked with 1% BSA/PBS for 1 h, rinsed with PBS, and incubated with anti-TAGLN2 antibody overnight at 4 °C. Secondary antibodies were added after washing and incubated in the dark for 1 h at room temperature. To visualize *E. coli* phagocytosis *in vitro*, GFP-expressing *E. coli* was generated by transformation with pGEX4T_GFP. Images were recorded every 10 s for a total of 900 s using an FV1000 laser scanning confocal microscope (Olympus, Tokyo, Japan). For live imaging in RAW 264.7 cells, 2 × 10^6^ polystyrene beads of diameter 4.5 μm (Polysciences Inc., Warrington, PA) were incubated in 10 mg/mL bovine serum albumin (BSA, Sigma) in PBS for 1 h at 4 °C. The beads were then washed 5 times in PBS and resuspended in 1 mL PBS. Rabbit anti-bovine albumin, IgG fraction (anti-BSA IgG, BD bioscience), was added at a final dilution of 1:500 and incubated for 30 min at 37 °C and 10 min at 4 °C. The beads were then washed thrice in 1 mL PBS.

### Reverse transcription quantitative (RT-q) PCR

Total RNA was isolated from cells or homogenized tissues of C57BL/6 mice with TRI Reagent (Molecular Research Center, Inc.) and reverse-transcribed using RT-Premix (iNtRON biotechnology). Primer sequence and PCR condition were described in Supplemental methods.

### Luciferase assay for NF-κB activity

Freshly isolated peritoneal macrophages were plated in 12 well plates the day before transfection and transfected with 1 µg of full-length of TAGLN2 reporter construct or 5′ deletion mutants with 1 µg of pRL-TK renilla and 1 µg of pGL3/NF-κB. At 48 h post-transfection, cell lysates were prepared and assayed for luciferase activity using the Dual Luciferase Assay System (Promega, Madison, WI), according to the manufacturer’s instructions.

### Ruffling assay

Cells were starved for 3 h and fMLP (200 nM) was added to the media. After 2 min, the cells were fixed, permeabilized, and stained with TRITC-phalloidin. Ruffling index was recored as described previously^46^. For time-lapse imaging of membrane dynamics, cells were grown on glass bottom tissue cultured plate and serum-starved for 3 h before stimulation with fMLP. Differential interference contrasts (DICs) were acquired without or with fMLP over 10 min with 10-s intervals using a confocal microscope (see Supplemental methods).

### Determination of cellular F-actin content

Macrophages were treated with LPS for 24 h and then the cells were fixed with 4% paraformaldehyde. Fixed cells were washed once with PBS and resuspended in PBS containing 1% BSA and 0.25% Triton X-100 for 5 min. After permeabilization, cells were washed, stained for 30 min with TRITC-phalloidin, and then analyzed by FV1000 confocal microscope (Olympus) or FACScanto flow cytometry (BD). In some experiments, LPS-stimulated macrophages (24 h) were further stimulated with 200 nM fMLP.

### *In vitro* and ***vivo*** phagocytosis assay

*In vitro* Fc- or CR-mediated phagocytosis was performed as previously described^47^ and detailed in Supplemental methods. To measure *in vivo* phagocytic activity, GFP-expressing *E. coli* (10^8^ CFU/mouse, DH5α) were injected into either wild-type or *TAGLN2* ^−/−^ mice i.p., and macrophages were harvested 2 h later.

### Sepsis and bacterial burden determination

For sepsis models with a single bacterial species, bacterial peritonitis was induced by i.p. injection of either 5 × 10^8^ CFU of live *E. coli* (DH5α) or 5 × 10^6^ CFU of live *S. typhimurium*. Survival was monitored once per 12-h period for 72 h. For determination of bacterial burden, thioglycollate-induced peritoneal macrophages (10^7^) obtained from wild-type or *TAGLN2* ^−/−^ mice were suspended in 200 μL PBS and injected i.p. into wild-type recipient mice with 1 × 10^8^ CFU of live *E. coli* (DH5α) bacteria. At 4 h post-injection, 5 mL of PBS was injected into the peritoneal cavity and peritoneal lavage fluid were diluted in sterile PBS, plated onto separate Luria-Bertani agar plates, and the plates were incubated for 24 h at 37 °C. Colonies were counted separately for each sample.

### Measurement of cytokines

Cytokines were measured from plasma using ELISA kits from R&D Systems, as per the manufacturer’s protocol. The levels of TNF-α, IL-1β, IL-6, and IL-12 were measured at the indicated times after injection of *S. typhimurium*.

### *In vivo* macrophage depletion and adoptive transfer

Macrophages were depleted using clodronate liposomes. Mice were injected i.p. with 200 μL of a suspension of clodronate liposomes or control liposomes 48 h before being infected with *E. coli* or *S. Typhimurium*. The depletion analysis was performed at 48 h after treatment by flow cytometry using F4/80 and CD11b staining. Clodronate liposome treatment resulted in an 85% decrease of macrophages in the spleen. Thioglycollate-induced peritoneal macrophages (10^7^) obtained from wild-type or *TAGLN2* ^−/−^ mice were suspended in 200 μL PBS and injected i.p. into clodronate liposome-treated recipient mice.

### Statistics

Mean values were calculated from at least three independent experiments unpaired Student’s t-tests, and one-way analysis of variance (ANOVA) tests were used to identify significant differences between multiple groups. A *P-*value < 0.05 was considered significant. Kaplan-Meier survival curves were calculated using the Prism software.

## Electronic supplementary material

Supplementary information

Supplementary Video 1

Supplementary Video 2

Supplementary Video 3

Supplementary Video 4

Supplementary Video 5

Supplementary Video 6

Supplementary Video 7

Supplementary Video 8
