# Supplementary material for: An Essential Role for TAGLN2 in Phagocytosis of Lipopolysaccharide-activated Macrophages
Source: Sci Rep. 2017 Aug 18;7:8731. doi: 10.1038/s41598-017-09144-x (PMC5562783; doi:10.1038/s41598-017-09144-x)
Supplement: Supplementary file 1 — Supplementary information [file 41598_2017_9144_MOESM1_ESM.pdf]

Supplementary Materials for

An Essential Role for TAGLN2 in Phagocytosis of Lipopolysaccharide-activated Macrophages

Hye-Ran Kim[1,2], Hyun-Su Lee[1,2], Kyung-Sik Lee[1,2], In Duk Jung[4], Min-Sung Kwon[1,2,5], Chang-Hyun Kim[1,2], Seong-Min Kim[3], Myung-Han Yoon[3], Yeong-Min Park[4], Sang-Myeong Lee[6], & Chang-Duk Jun[1,2,*]

This PDF file includes:

Figs. S1 to S9

Figure legends for Figs. S1 to S4

Captions for Video 1 to 8

Supplemental Materials and Methods

Figure S1

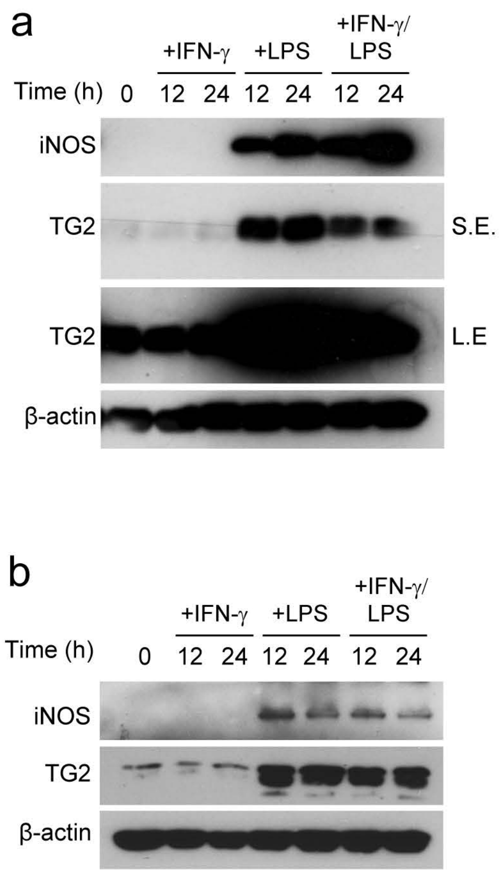
a
+IFN-γ
+LPS
+IFN-γ/
LPS
Time (h)
0
12
24
12
24
12
24
iNOS
TG2
S.E.
TG2
L.E
β-actin
b
+IFN-γ
+LPS
+IFN-γ/
LPS
Time (h)
0
12
24
12
24
12
24
iNOS
TG2
β-actin

Figure S2

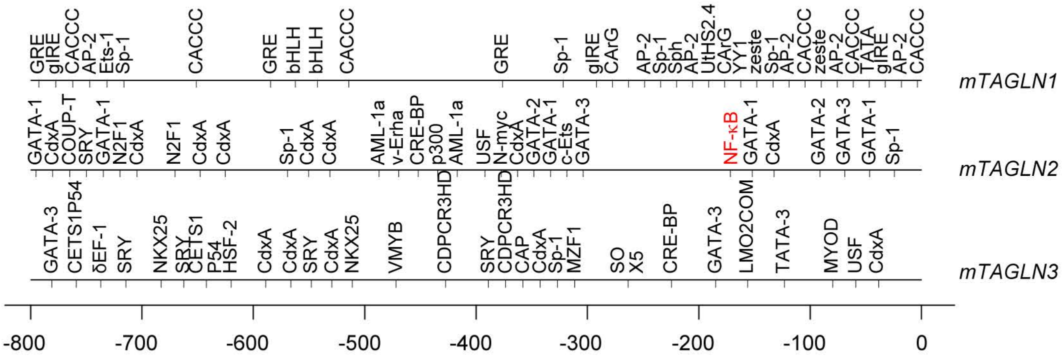
mTAGLN1
mTAGLN2
mTAGLN3
GRE
gIRE
CACCC
AP-2
Ets-1
Sp-1
bHLH
CArG
Sph
UtHS2.4
YY1
zeste
TATA
GATA-1
CdxA
COUP-T
SRY
N2F1
AML-1a
v-Erha
CRE-BP
p300
USF
N-myc
GATA-2
c-Ets
GATA-3
NF-κB
CETS1P54
δEF-1
NKX25
CETS1
P54
HSF-2
VMYB
CDPCR3HD
CAP
MZF1
SO
X5
LMO2COM
TATA-3
MYOD
-800
-700
-600
-500
-400
-300
-200
-100
0

Figure S3

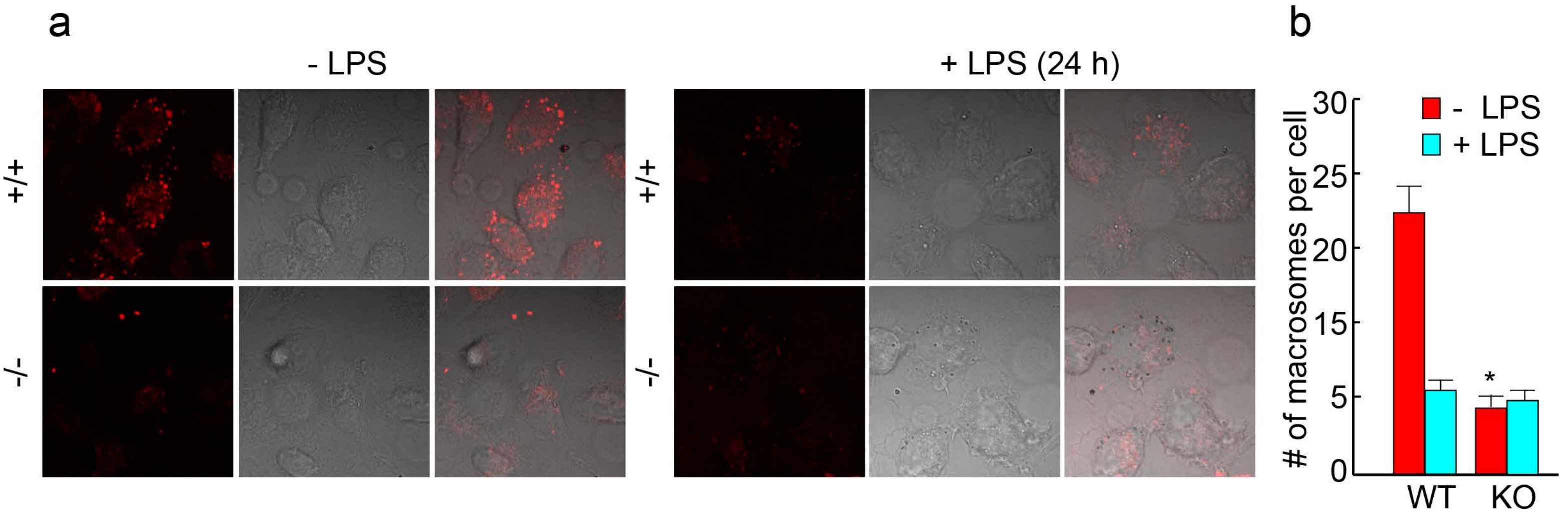
a
- LPS
+ LPS (24 h)
+/+
-/-
b
# of macrosomes per cell
30
25
20
15
10
5
0
- LPS
+ LPS
*
WT
KO

Figure S4

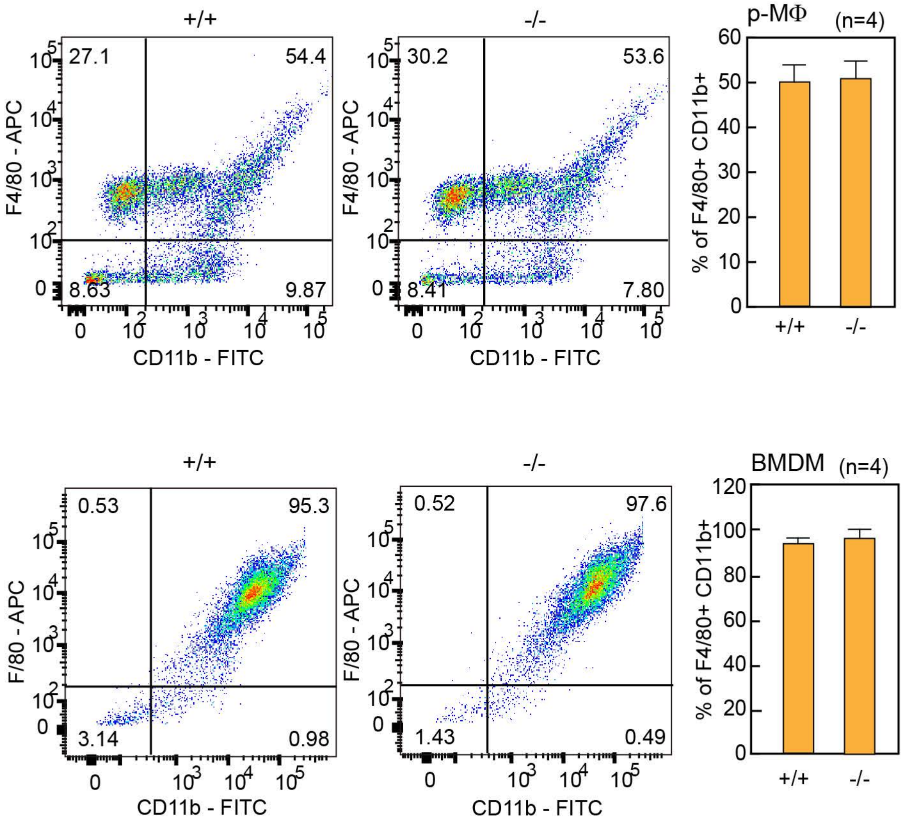
+/+
-/-
p-MΦ
(n=4)
27.1
54.4
8.63
9.87
30.2
53.6
8.41
7.80
F4/80 - APC
CD11b - FITC
% of F4/80+ CD11b+
0.53
95.3
3.14
0.98
0.52
97.6
1.43
0.49
F/80 - APC
BMDM
(n=4)

Figure S5

Figure 1a

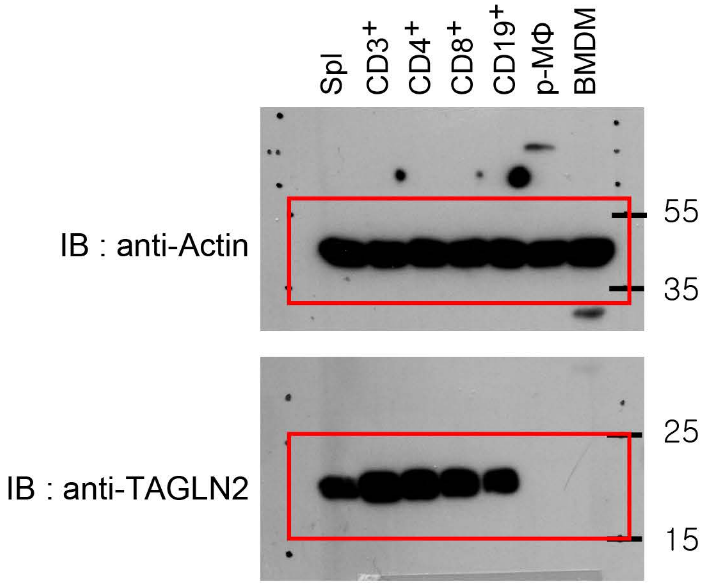

Figure 1b

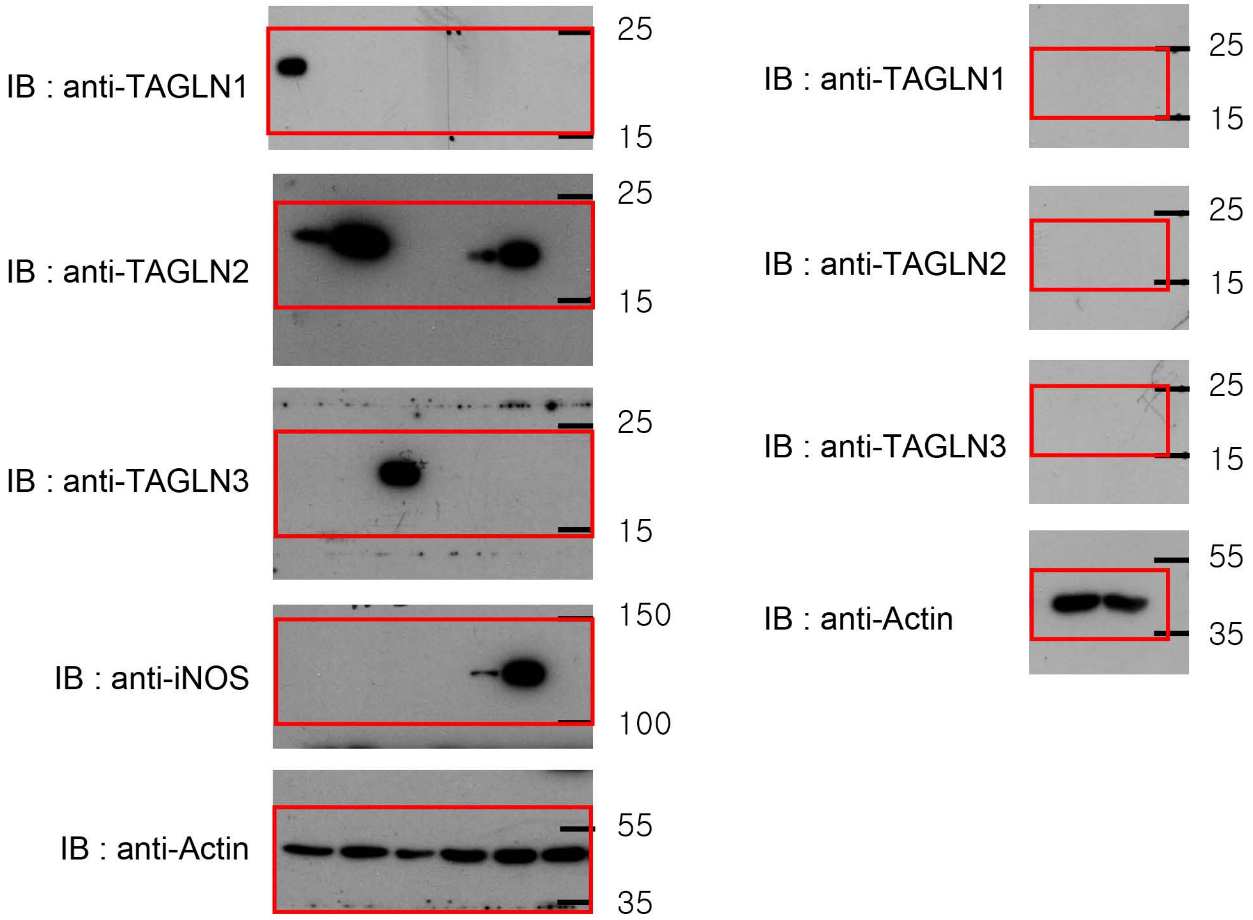

Figure S6

Figure 1c

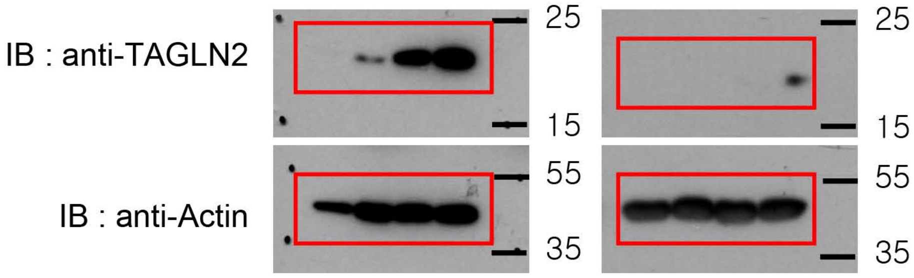

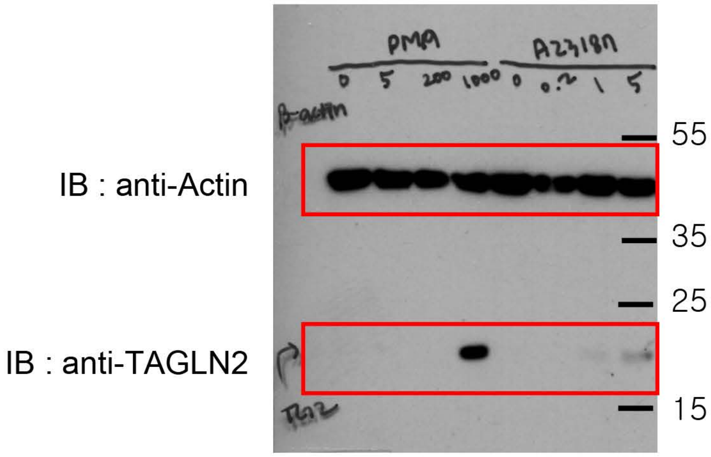

Figure 1d

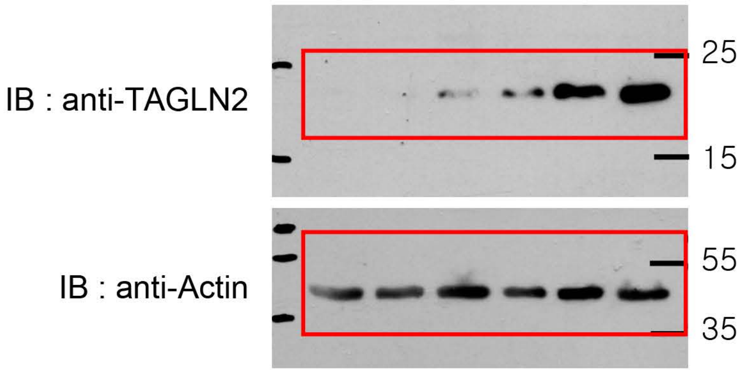

Figure S7

Figure 1e

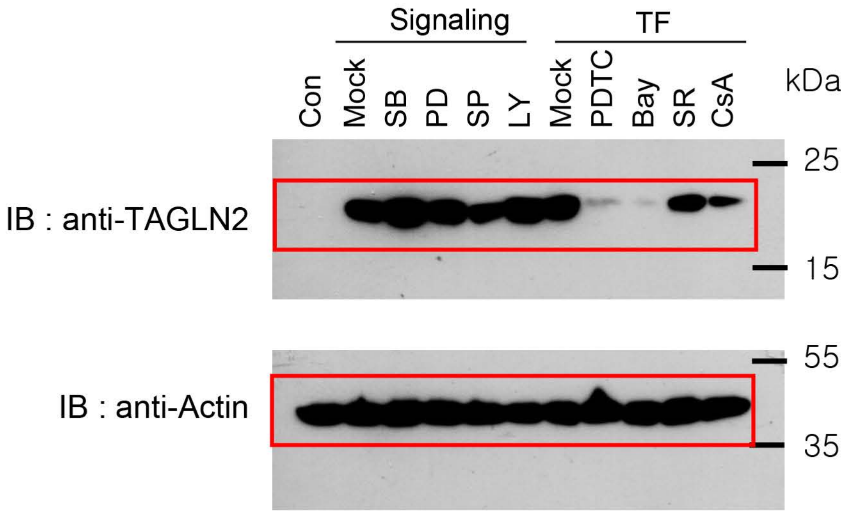

Figure S8

Figure 2a

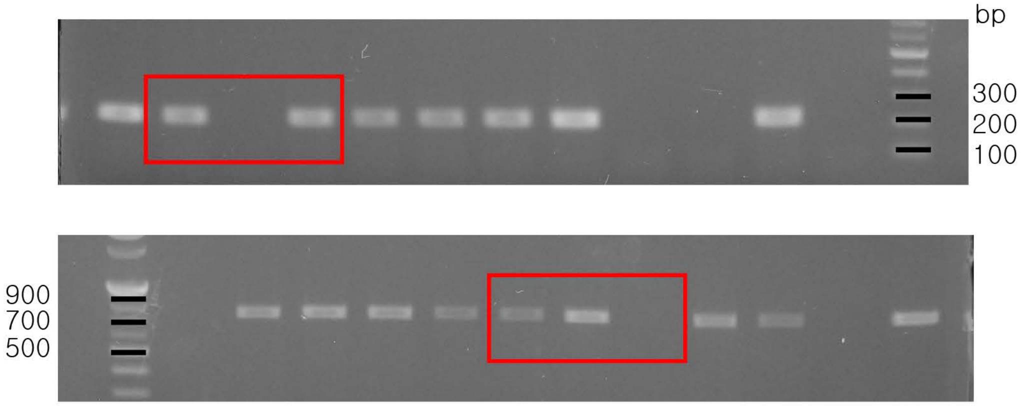

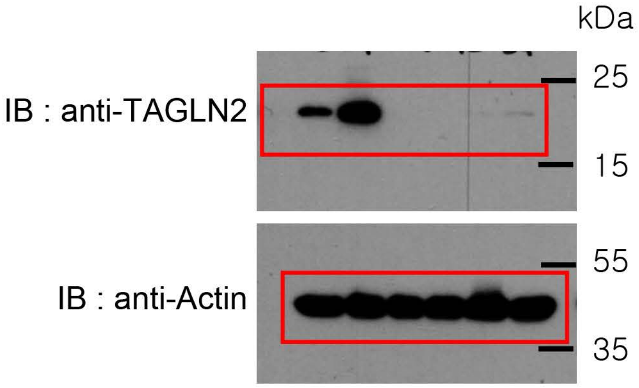

Figure S9

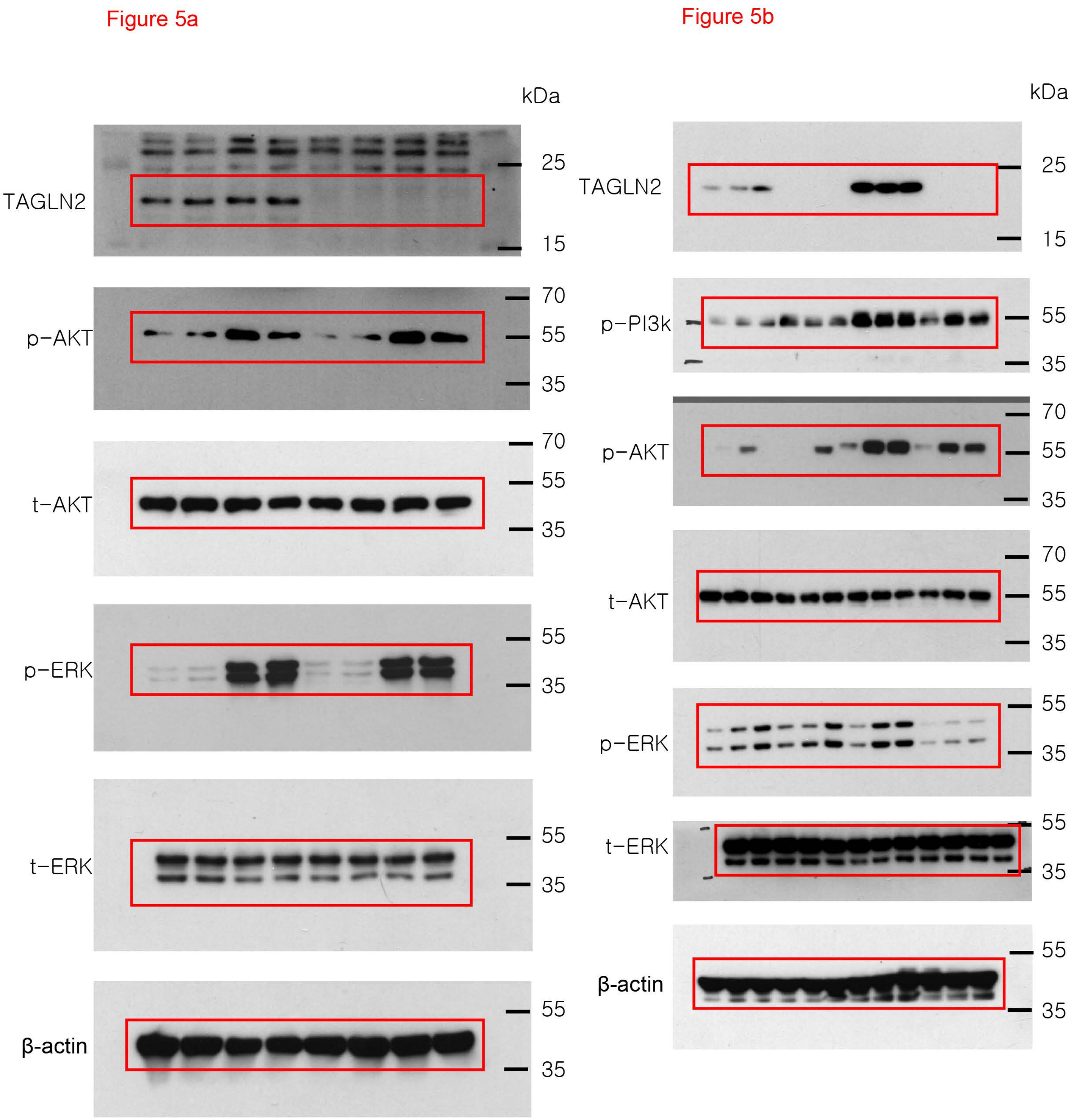

Figure 5a
kDa
TAGLN2
25
15
p-AKT
70
55
35
t-AKT
70
55
35
p-ERK
55
35
t-ERK
55
35
β-actin
55
35
Figure 5b
kDa
TAGLN2
25
15
p-PI3k
55
35
p-AKT
70
55
35
t-AKT
70
55
35
p-ERK
55
35
t-ERK
55
35
β-actin
55
35

Figure S1. Detection of TAGLN2 in peritoneal and bone marrow-derived macrophages with short- and long-time exposure.

Peritoneal (a) and bone marrow-derived (b) macrophages were isolated from wild-type mice and activated with indicated stimulants. IFN-γ (100 ng/ml), LPS (1 μg/mL). Cell lysates were subjected to western blot analysis using antibodies against transgelin-2. iNOS expression was used as a positive control. Protein, 30 μg; S.E, short exposure, time: 1 min; L.E, long exposure, time: 10 min.

Figure S2. Analysis of mouse TAGLN-1, 2, and 3 promoter region.

Schematic diagram of the promoter region of TAGLN family. Transcription factor binding sites were identified using the computer program Factor (HUSAR program package, DKFZ Heidelberg). Note the presence of NF-κB binding site in TAGLN2 only.

Figure S3. Defective macropinocytosis in resting macrophages from *TAGLN2*$^{-/-}$ mice

(a) Peritoneal macrophages from wild-type and *TAGLN2*$^{-/-}$ mice were cultured with or without LPS (1 μg/mL) for 24 h. Cells were then incubated with 500 μg/ml of lysine-fixable, Texas Red-conjugated 70 kDa dextran for 15 min and imaged under confocal microscopy. Scale bars, 10 μm. (b) The number of macropinosomes per cells were analyzed by ImageJ as described in Supplementary Methods. *$P < 0.05$ *vs*. wild-type resting macrophages.

Figure S4. Development and activation of macrophages obtained from wild-type and *TAGLN2*$^{-/-}$ mice. Thioglycollate-induced peritoneal (P-MΦ) and bone marrow-derived (BMDM) macrophages obtained from wild-type and *TAGLN2*$^{-/-}$ mice were stained with antibodies against CD11b^{+} and F4/80^{+}, and then, the cells were analyzed by flow cytometry.

The dot plots shown are representative of 4 experiments, each using a separate mouse. The percentage of $CD11b^+$ and $F4/80^+$cells was compared between wild-type and *TAGLN2*$^{-/-}$ mice.

Video S1. Peritoneal macrophages from wild-type mice were cultured for 24 without LPS and GFP- expressing *E. coli* (2×10^6) were added before imaging. Time-lapse images were taken at 10-s intervals for 15 min. This video corresponds to Fig. 2d.

Video S2. Peritoneal macrophages from *TAGLN2*$^{-/-}$ mice were cultured for 24 without LPS and GFP- expressing *E. coli* (2×10^6) were added before imaging. Time-lapse images were taken at 10-s intervals for 15 min. This video corresponds to Fig. 2d.

Video S3. Peritoneal macrophages from wild-type mice were stimulated with LPS (1 μg/mL) for 24 h and GFP- expressing *E. coli* (2×10^6) were added before imaging. Time-lapse images were taken at 10-s intervals for 15 min. This video corresponds to Fig. 2d.

Video S4. Peritoneal macrophages from *TAGLN2*$^{-/-}$ mice were stimulated with LPS (1 μg/mL) for 24 h and GFP-expressing *E. coli* (2×10^6) were added before imaging. Time-lapse images were taken at 10-s intervals for 15 min. This video corresponds to Fig. 2d.

Video S5. RAW 264.7 cells were co-transfected with TG2_GFP and LifeAct, as described in the legend for Fig. 3e. IgG-opsonized beads were added 5 min before acquiring confocal images every 20 sec for 10 min.

Video S6. Peritoneal macrophages from wild-type mice were stimulated with LPS (1 μg/mL) for 24 h and serum-starved for 3 h before fMLP (200 nM) stimulation. Time-lapse images of

phase-contrast microscopy were taken at 10-s intervals for 10 min (FV1000; Olympus). This video corresponds to Fig. 4b.

Video S7. Peritoneal macrophages from *TAGLN2*$^{-/-}$ mice were stimulated with LPS (1 μg/mL) for 24 h and serum-starved for 3 h before fMLP (200 nM) stimulation. Time-lapse images of phase-contrast microscopy were taken at 10-s intervals for 10 min (FV1000; Olympus). This video corresponds to Fig. 4b.

Video S8. RAW 264.7 cells were transfected with TAGLN2_GFP and LifeAct and then treated with 100 nM PMA. Live cell imaging was performed on a confocal microscope (FV1000; Olympus). Images were acquired every 30 sec for 10 min.

Supplementary Methods

Reagents and antibodies

Rabbit polyclonal anti-TAGLN2 antibody was raised in rabbits using purified full-length TAGLN2 (AbFrontier, Seoul, Korea). The following antibodies were used: goat polyclonal anti-TAGLN1 (Santa Cruz Biotechnology, Dallas, TX, USA); rabbit polyclonal anti-β-actin, rabbit polyclonal antibodies against p-ERK, ERK, p-JNK, JNK, p-PI3K, PI3K, p-AKT, AKT, and HRP-conjugated anti-mouse IgG, anti-goat IgG, and anti-rabbit IgG (Cell Signaling Technology, Danvers, MA, USA); mouse monoclonal anti-TAGLN3 (Abcam, Cambridge, MA, USA); FITC-conjugated anti-CD11b and APC-conjugated anti-F4/80 (eBioscience, San Diego, CA, USA); and anti-mouse CD28 (R&D Systems, Minneapolis, MN, USA). TRITC-phalloidin, poly-L-lysine (PLL), A23187, phorbol 12-myristate 13-acetate (PMA), SB 431542, PD0325901, SP600125, LY294002, ammonium pyrrolidinedithiocarbamate (PDTC), Bay117082, SR11302, and Cyclosporin A (CsA) were purchased from Sigma-Aldrich (St. Louis, MO, USA). Lipofectamine 2000 transfection reagent was purchased from Life Technologies (Carlsbad, CA, USA). Recombinant IFN-γ was obtained from Peprotech Inc. (Rocky Hill, NJ). LPS, fMLP (*N*-formyl-Met-Leu-Phe), sodium nitrite, 85% phosphoric acid, and *N*-(1-naphthyl) ethylenediamine dihydrochloride were purchased from Sigma Aldrich. Duo Set mouse IL-12, IL-6, IL-1β, and TNF-α ELISA kits were obtained from R&D system. Sheep RBCs were purchased from Hanil KOMED (Seoul, Korea). Anti-Sheep RBC IgG and IgM antibodies were purchased from MP Biomedicals (Solon, OH, USA). Conventional PCR premix and SYBR were obtained from Enzynomics (Korea). Clondronate and control liposome for macrophage depletion were purchased from FormuMax (Sunnyvale, CA, USA). *E. coli* strain DH5α was purchased from ATCC and *Salmonella Typhimurium* was a gift from Dr. Sang-Myong Lee (Jeonbuk National University).

Reverse transcription quantitative (RT-q) PCR

PCR was performed with the following primers (the respective forward and reverse pairs are indicated): mouse *GAPDH*, 5′-GCACAGTCAAGGCCGAGAAT-3′ and 5′-GCCTTCTCCATGGTGGTGAA-3′; mouse *TAGLN1*, 5′-ATGTTCCAGACTGTTGACCT-3′ and 5′-ACTGATGATCTGCCGGGGTC-3′; mouse *TAGLN2*, 5′-TCTTTGCCATCACCACAGCTGCTCAGAATG-3′ and 5′-CGTGCCGTCCTTGAGCCACTTCTGGAAGTT-3′; mouse *TAGLN3*, 5′-GGTCCTGTGCAAGCTGATAAA-3′ and 5′-GGGCTTTCCTGTGAAACCAG-3′. The thermal cycling conditions consisted of 30 cycles of denaturation at 94°C for 30 s, annealing at 60–62°C for 20 s, and extension at 72°C for 40 s preceded by denaturation at 94°C for 7 min. The expression levels of the mouse *TAGLN1, 2,* and *3* were evaluated by RT-qPCR, unless otherwise indicated. Amplification was performed in StepOne Real-Time PCR Systems (Applied Biosystems) for continuous fluorescence detection in a total volume of 10 µL of cDNA/control and gene-specific primers by using SYBR Premix Ex *Taq* (Takara Bio). The mRNA levels of the target genes, relative to *GAPDH*, were normalized using the following formula: relative mRNA expression = $2^{-(\Delta\mathrm{Ct\ of\ target\ gene}\ -\ \Delta\mathrm{Ct\ of\ GAPDH})}$, where Ct is the threshold cycle value. In each sample, the expression of the analyzed gene was normalized to that of *GAPDH* and described as the mRNA level relative to *GAPDH*.

Scanning electron microscopies

For scanning electron microscopy (SEM), cells were fixed with 2.5% glutaraldehyde solution in sodium cacodylate for 1 h at 4°C, rinsed with sodium cacodylate for 5 min, and fixed in OsO_4 for 20 min. Then, the samples were dehydrated through incubation with a graded

ethanol series over 30 min and dried in a critical point dryer. Samples were prepared by sputter coating with 1–2 nm gold-palladium and analyzed using FE-SEM (HITACHI, Tokyo, Japan).

Western blot analysis

Cells or homogenized tissues from C57BL/6 mice were lysed in ice-cold lysis buffer (50 mM Tris-HCl pH 7.4, 150 mM NaCl, 1% Triton X-100, 1X complete protease/phosphatase inhibitor cocktail) for 1 h on ice. Cell lysates were centrifuged at 16,000 × *g* for 25 min at 4°C. The supernatants mixed with SDS sample buffer (100 mM Tris-HCl pH 6.8, 4% SDS, 20% glycerol, bromophenol blue) and heated for 5 min. Proteins were separated by 12% SDS-PAGE and transferred to nitrocellulose membranes using a Trans-Blot SD Semi-Dry transfer cell (Bio-Rad, Hercules, CA, USA). The membranes were blocked in 5% skim milk for 1 h, rinsed, and incubated with primary antibodies in TBS containing 0.1% Tween-20 (TBS-T) and 3% milk overnight. Excess primary antibody was removed by washing the membrane four times in TBS-T before incubation with peroxidase-labeled secondary antibody (0.1 μg/mL) for 1.5 h. Bands were visualized with a western blot detection kit (Intron Biotechnology, Seongnam, Korea) and exposed to X-ray film.

Ruffling assay

Cells were grown on glass coverslips 24 h without FBS before ruffling studies. For macrophage stimulation, fMLP was added to the media to a final concentration of 200 nM and after 2 min, the cells were fixed with 3.7% PFA for 10 min at room temperature, permeabilized with 0.1% Triton X-100, and stained with TRITC-phalloidin. Then, the ells were analyzed under a confocal microscope. As described previously [47], ruffling was defined by the presence of F-actin–rich submembranous folds. The cells were scored on a scale of 0–

2, where 0 indicates no ruffles, 1 indicates ruffling confined to one half of the cell dorsal surface, and 2 indicates ruffling over the entire dorsal surface. For each condition, 100 macrophages were analyzed and the ruffling index was recorded as the sum of the ruffling scores of 100 cells.

For time-lapse imaging of membrane dynamics, peritoneal macrophages were grown on glass bottom tissue cultured plate and serum-starved for 3 h before stimulation with fMLP. The cells were placed in a live-imaging chamber filled with 5% CO_2 and mounted in a temperature-controlled stage heater set at 37 °C on an inverted microscope. Differential interference contrasts (DICs) were acquired without or with fMLP over 10 min with 10-s intervals using a confocal microscope. HEPES buffer (137 mM NaCl, 5 mM KCl, 1 mM sodium phosphate, 6 mM glucose, 1 mM $CaCl_2$, 0.5 mM $MgCl_2$, 1% BSA, and HEPES 10 mM pH 7.3) was used in place of PBS for live-cell imaging. For image analysis, one pixel line was drawn along the cell membrane and kymographs were generated using ImageJ. Quantitative parameters derived from kymographs were calculated as previously described [48]. Briefly, a one pixel-wide line crossing the edge of the cell perpendicularly at the location of the protrusion was traced. This line records protrusion dynamics relative to the substratum since the position of the region of interest (the line) was fixed. Next, the snapshots corresponding to the line were lined up according to the sequence of image acquisition. The resulting images were displayed on a computer screen and shades of gray were used to identify protruding and retracting segments by manually drawing ascending and descending lines with a computer mouse using ImageJ segmented line tool. Raw data were exported to Excel to calculate protrusion/retraction persistence and velocity, and ruffling frequency. For the co-localization study in live cells, RAW 264.7 cells were cultured and transiently transfected with either GFP-tagged TAGLN2 plasmid or LifeAct_mCherry. On the day of analysis, the cells were placed on a glass-bottomed confocal dish and the TAGLN2_GFP-

and LifeAct-positive cells were imaged in a HEPES live-cell imaging buffer (137 mM NaCl, 5 mM KCl, 1 mM sodium phosphate, 6 mM D-glucose, 1 mM $CaCl_2$, 0.5 mM $MgCl_2$, 1% BSA, and 10 mM HEPES; pH 7.3) at 37 °C using the 100× NA 1.40 oil immersion objective of a laser-scanning confocal microscope (FV1000; Olympus). Time-lapse and z-stack images were collected every 20 s for 10 min at 37 °C. The data were collected, processed, and analyzed using the FLUOVIEW software (Olympus).

Phagocytosis assay

Sheep RBC (sRBC) were pelleted and washed three times with PBS. sRBC were opsonized with rabbit anti-sheep RBC IgG diluted 1:100 (MP Biomedicals) and incubated at room temperature with gentle rotation for 30 min. C3bi opsonization was performed by first incubating sRBC with sub-agglutinating concentrations of IgM (1:10) in PBS with 0.5 mM $CaCl_2$ and $MgCl_2$ for 30 min at 37°C. Excess IgM was washed off, and sRBC were incubated with C5-deficient serum (5% v/v) for 30 min at 37°C with frequent mixing. Opsonized sRBC were washed three times and resuspended in PBS. The cells were counted and diluted so that the sRBC/macrophage ratio was 10:1. Macrophages were chilled on ice before addition of the opsonized sRBC, and plates containing macrophages and sRBC were spun down for 1 min (1000 rpm). The plates were incubated on ice for 10 min, medium was then aspirated, and warm medium was added to initiate phagocytosis. The plates were incubated at 37°C for the indicated times. Reactions were stopped by placing them on ice and washing away uningested particles with ice-cold PBS three times, followed by hypotonic lysis of any remaining uningested sRBC with water for 1 min. The cells were washed an additional three times with PBS followed by fixation with 2.5% fresh, cold glutaraldehyde for 30 min. sRBC were visualized by light microscopy. The phagocytic index was calculated by selecting 5–10 microscope fields and scoring each field for both total number of macrophages and total

number of ingested targets. A minimum of 100 macrophages was scored per coverslip. The phagocytic index indicates the number of particles ingested by 100 cells.

Fluid phase uptake assays

Freshly isolated peritoneal macrophages were cultured with or without LPS (1 µg/mL) for 24 h and incubated with 500 µg/ml of lysine-fixable, Texas Red-conjugated 70 kDa dextran (Molecular Probes, Eugene, OR, USA) for 15 min at 37°C in serum-free RPMI. Following the incubation, cells were placed on ice for 5 min to inhibit further uptake of dextran, washed with cold PBS, and fixed with 4% paraformaldehyde. Images were taken using an FV1000 confocal microscope and quantified for macropinosomes using an automated image analysis pipeline in ImageJ (NIH). The 'Substract Background' functionality was executed, and images thresholded to a binary image. Dextran-positive structures at least 0.5 µm in diameter were quantified through the 'Analyze Particles' feature.
